# Signaling pathways and targeted interventions for precancers

**DOI:** 10.1038/s41392-025-02342-4

**Published:** 2026-01-07

**Authors:** Jin Yang, Shimeng Wang, Xin Li, Hongdan Xu, Tongxu Sun, Tao Hu, Jingjing Luo, Hongmei Zhou

**Affiliations:** 1 State Key Laboratory of Oral Diseases, National Center for Stomatology, National Clinical Research Center for Oral Diseases, Frontier Innovation Center for Dental Medicine Plus, West China Hospital of Stomatology, Sichuan University, Sichuan, PR China; 2 Department of Pathology and Laboratory Medicine, School of Medicine, University of California, Davis, Sacramento, CA USA

**Keywords:** Therapeutics, Cancer

## Abstract

Precancers, defined as normal-appearing or morphologically altered tissues with a risk of oncogenesis, exhibit various detectable manifestations across anatomical sites, including epithelial dysplasia, metaplasia, hyperplasia, and stromal fibrosis. Considering the prevailing assumption that most cancers arise from precancers, early intervention at the precancerous stage has immense potential to reduce cancer-related morbidity and mortality. However, the complex signaling networks governing precancer initiation and progression remain elusive, hampering the development of effective targeted interventions. This review synthesizes three critical dimensions of precancer biology: historical foundations tracing the conceptual evolution of precancer research over the past century; mechanisms underlying the multistep progression of precancer biology, encompassing epithelial and macro/microenvironmental remodeling; and signaling networks cataloging dysregulated pathways and their therapeutic potential. Over 10 signaling pathways, including the transforming growth factor-β (TGF-β), p53, Wnt, phosphatidylinositol 3-kinase (PI3K), and mitogen-activated protein kinase (MAPK) pathways, drive multistep malignant transformation. We further synthesize emerging evidence supporting microenvironmental dominance, proposing the novel “soil degeneration” hypothesis. This paradigm shift underscores the necessity for dual-window intervention in which early-phase microenvironmental normalization prevents the establishment of precancerous lesions and advanced-phase treatment concurrently addresses epithelial malignancy and stromal degeneration. This review bridges foundational molecular discoveries with translational clinical potential and advocates for precision intervention frameworks that extend from biomarker-guided risk assessment to synergistic remodeling of the precancer microenvironment, thereby redefining precancer intervention in the molecularly targeted era.

## Introduction

Precancers encompass any condition, state, or lesions characterized by molecular signatures and histopathological alterations that confer malignant transformation potential.^1,2^ These transitional states demonstrate progressive genomic instability, microenvironmental remodeling, and dysregulated signaling pathways, which fundamentally differentiate them from normal tissue homeostasis while predisposing them to malignant progression. The evolving concept of “precancer”, formally adopted by the United States National Cancer Institute,^2,3^ has expanded to include epithelial, lymphoid, hematologic, and soft tissue precancers, as well as entities diagnosed through nonmorphologic methods.^3^ Current research trends, as evidenced by bibliometric analysis of the past decade on precancers, demonstrate a predominant emphasis on epithelial-origin lesions—a focus concordant with epidemiological data indicating that epithelial-derived malignancies account for approximately 80% of human cancers.^4^ In addition, precancers have been well characterized in hematological malignancies.^5^

Precancerous interventions assume critical importance given current cancer epidemiology: the 2022 global cancer statistics reached 20 million cancer cases with 9.7 million deaths,^6^ accounting for 1 in 6 global deaths (the second leading cause of mortality).^6–8^ Paradoxically, while the prevailing assumption suggests that the majority of cancers arise from identifiable precancers,^9–16^ almost 50% of cancers still present with locally advanced or metastatic disease at diagnosis.^17,18^ This diagnostic latency underscores the imperative for secondary prevention strategies targeting the premalignant phase. Precancers create observable windows of susceptibility, wherein individuals exposed to detrimental insults demonstrate a significantly elevated risk of malignant progression compared with their normal counterparts.^19,20^ The therapeutic advantage of precursor lesions—characterized by greater susceptibility to treatment and less heterogeneity than cancers—establishes precancers as high-value targets for interception strategies. The clinical benefits are validated by the substantial decline in cervical cancer incidence following the implementation of population-based HPV vaccination and cytologic screening.^21,22^ Similarly, the presence of favorable prognostic parameters in oral cancers with documented dysplastic history,^23,24^ and the significant reduction in the incidence and mortality of upper gastrointestinal cancers following one-time endoscopic screening,^25^ further underscore the importance of precancerous interventions.

Deciphering the molecular underpinnings of precancer biology will lay the foundation for developing clinically impactful and broadly applicable intervention strategies. We argue that research in this field should prioritize three key objectives: understanding the intrinsic and environmental features that distinguish high-risk lesions from those likely to regress; systematically mapping dysregulated signaling cascades along malignant transformation trajectories before heterogeneous cancers arise; and identifying generalizable and actionable interventions at precancer stage. While emerging studies have begun characterizing premalignant molecular landscapes, knowledge gaps persist in comprehensively understanding pathway dysregulation and developing corresponding targeted therapies. In light of this, the present review synthesizes cross-disciplinary evidence from molecular pathogenesis studies to clinical trials, with particular emphasis on a dual-window intervention paradigm: early-phase interventions aimed at microenvironmental normalization to prevent the establishment of precancerous lesions and advanced-phase interventions employing combinatorial therapies that simultaneously target epithelial mutations and microenvironmental drivers. This approach leverages the therapeutic window before irreversible malignant transformation, offering a strategic framework for precision prevention and treatment.

## Historical context of precancer research

**Fig. 1:**
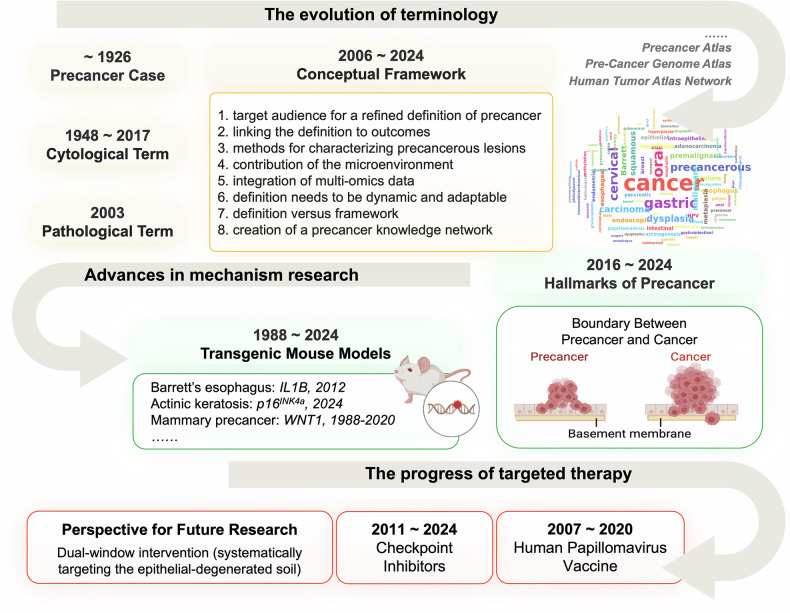
Historical Overview of Precancer Research. Research on precancers can be divided into three main stages: (1) the terminology evolution of precancers and related diseases (yellow box); (2) advancements in mechanism research (green box); and (3) key molecular events related to therapeutic approaches (red box)

Understanding precancers has undergone a long and intricate journey, primarily encompassing three stages: term evolution, advancements in mechanistic research, and the progress of targeted therapy (Fig. 1), which present the key milestones that represent significant shifts in precancer research.

### The evolution of terminology

The broad concept of “precancers”—identifiable diseases that precede cancer development—first emerged in clinical reports traceable to 1926 in the Web of Science.^26^ Despite nearly a century of observation, the field continues to grapple with fundamental challenges in establishing unified research paradigms across organ systems, particularly for multiple medical discipline communities. Scholars have attempted to classify precancers at both the cytological and pathological levels. Dr. Ayre J. E. used the term “precancer” in 1948 to describe cellular malignant progression, alongside normal, inflammatory, and cancer cells.^27^ In 2015, a working conference on the premalignant phases of neoplastic disorders proposed a more refined cellular terminology, distinguishing between normal and reactive cells in a physiological state and clonal/monoclonal, premalignant neoplastic, and cancer cells in a pathological state.^28^ From a pathological perspective, Berman and Henson introduced a metadata-based approach to categorize six precancer classes: acquired microscopic lesions, acquired macroscopic lesions with microscopic atypia, inherited hyperplastic syndromes that could progress to cancer, acquired diffuse hyperplasia/metaplasia, currently unclassified entities, and superclasses and modifiers.^29^ However, neither of these classifications has been widely adopted. To support research, the United States National Cancer Institute recently proposed a conceptual framework for defining precancers, which includes several critical components, such as methods for characterization, the contribution of the microenvironment, the integration of multiomics data, and more.^2^ Overall, precancers are fundamentally differentiated from transient reactive changes by their persistent abnormalities and evolutionary trajectories, but they have not yet reached the uncontrollable states observed in cancer.

### Advances in mechanism research

In a recent review, Neelendu Dey, Ken S. Laug, and colleagues outlined six hallmarks of precancers, including age-associated genetic instability (telomere shortening and somatic mutations); locus-specific epigenetic dysregulation; metabolic alterations; hijacking of regenerative cell state transitions; immune-editing disruption and the senescent secretome (“inflammaging”); and senescent fibroblast-mediated microenvironmental remodeling.^1^ While these six characteristics capture several key biological dimensions, multiomics investigations, particularly through initiatives such as the Precancer Atlas (PCA) and Precancer Genome Atlas (PCGA),^30,31^ reveal additional molecular events requiring integration into the hallmarks of precancers, including genomic and epigenetic variations,^32,33^ microbiome‒host crosstalk,^34,35^ metabolomic rewiring,^36^ the precancer microenvironment (PME) complexity,^37–39^ etc. Despite these precancerous features exhibiting substantial overlap with established cancer hallmarks, the intact basement membrane serves as a clear biological boundary distinguishing precancers from cancers.^40^

Additionally, the development of animal models has advanced research on precancers, with two principal modeling strategies emerging, including chemical carcinogenesis and genetically engineered models. Most precancer models utilize traditional environmental carcinogens to mirror stepwise pathogenesis, such as 3′-methyl-4-dimethylaminoazobenzene (3′-Me-DAB)-induced hepatic precancerous lesions,^41^ 12-O-tetradecanoylphorbol-13-acetate (TPA) and 7,12-dimethylbenz[a]anthracene (DMBA) for actinic keratoses,^42^ and 4-nitroquinoline-1-oxide (4NQO)-mediated oral/esophageal tumorigenesis.^43^ Some precision precancerous models target driver pathways in tissue-specific contexts, such as the *IL1β* and *Kras* transgenic mouse model in Barrett’s esophagus (BE),^44,45^ knockout of *p16*^INK4a^ along with doxycycline exposure in actinic keratosis,^46^ and the MMTV-Wnt1 transgenic mouse model for mammary precancerous lesions.^20,47^ These complementary approaches enable stage-specific research on premalignant biology: chemical carcinogen models recapitulate cancer development timelines through serial longitudinal sampling, whereas genetic systems facilitate precise analysis of oncogenic pathways via inducible driver activation. However, for some less-studied cancers, faithful models have yet to be developed. CRISPR-Cas9-based genome editing may greatly expedite the generation of precancerous models.

### Progress in targeted therapy

The discovery of macro/microenvironmental factors has led to the catalysis of intervention transformation in precancer patients, prominently exemplified by human papillomavirus (HPV) and immune checkpoint modulation. HPV vaccination is effective in preventing HPV16/18-associated cervical intraepithelial neoplasia (CIN) and cancer among HPV-naïve and sexually active populations.^48^ The synthetic plasmid vaccine VGX-3100, which targets the E6/E7 proteins of HPV-16/18, triggers CIN regression.^49,50^ In addition, emerging clinical evidence positions precancers as an immunologically active window for checkpoint intervention. Anti-PD-1 immune checkpoint inhibitors have achieved a clinical response in oral and cervical precancers,^51,52^ which is attributed to the upregulated expression of programmed cell death ligand 1 (PD-L1) on premalignant cells.^53^ This precancer-targeted intervention evolution underscores the potential for intercepting malignant transformation through precision microenvironment remodeling rather than through conventional cytotoxic approaches.

## From normal to precancer and cancer

### The multistep malignant transformation process

The initiation of precancer and its progression to oncogenesis is generally a multistep process.^54,55^ In normal tissue, somatic mutations arise sporadically and are eliminated to maintain homeostasis. However, some oncogenic mutations escape tumor-suppressive mechanisms to confer clonal advantages (clonal/monoclonal cells). Additional exposure allows these clones to expand and accumulate further alterations (premalignant cells). Subsequent genetic mutations, epigenetic alterations, and the influence of a tumor-promoting microenvironment ultimately lead to irreversible transformation and highly heterogeneous lesions (cancer cells).

**Fig. 2:**
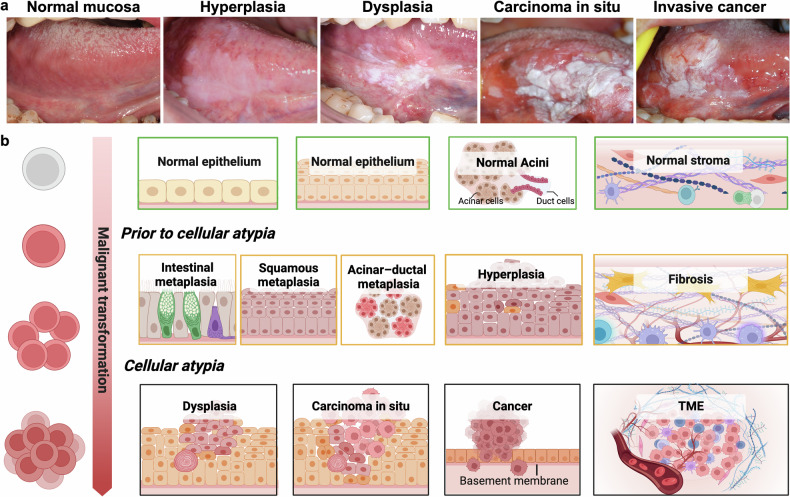
Main Histopathological Changes in Precancers. **a** Clinical progression of oral mucosal lesions: representative images illustrate the transition from normal mucosa to precancerous and malignant stages. Written informed consent was obtained from all participants prior to image acquisition. **b** During malignant transformation, oncogenic mutations provide clonal advantages (clonal/monoclonal cells), enabling these clones to expand and accumulate further alterations (premalignant cells), ultimately leading to irreversible transformation and highly heterogeneous lesions (cancer cells). Specifically, the ability to invade the basement membrane of tissue serves as a clear demarcation between precancers and cancers. Precancers at high risk manifest as epithelial dysplasia before carcinoma. Before cellular atypia, some histopathological changes, including intestinal metaplasia, squamous metaplastic, acinar–ductal metaplasia, hyperplasia, and fibrosis, can be observed in certain precancers. TME tumor microenvironment

Oral precancers serve as a paradigmatic model for macroscopic examination and are characterized by clinically detectable, progressive morphological changes in mucosal tissues (Fig. 2a). Multiple stages of the multistep sequence can even coexist within a single lesion, as evidenced by our previous findings.^56^ However, significant disparities exist in the criteria among different precancerous conditions. The absence of standardized terminology, particularly for in situ lesions across various anatomical sites, poses substantial challenges in establishing a unified research framework. To enhance the understanding of precancers and facilitate the integration of biological and clinical research, we present a comprehensive discussion of the principal histopathological features common to precancers (Fig. 2b).

#### Premalignant cellular atypia

The malignant potential of premalignant cells is driven primarily by the accumulation of oncogenic mutations, which confer clonal advantages and ultimately reach a tipping point where lesions irreversibly progress toward malignancy. Premalignant cells commonly exhibit cytologic atypia, characterized by features such as nuclear pleomorphism, prominent nucleoli, an enlarged nuclear‒cytoplasmic ratio, and increased mitotic activity, among others.^57^ However, these morphological alterations are generally less severe than those observed in fully malignant cells.

In solid tumors, cytologic atypia is frequently associated with structural epithelial abnormalities, collectively termed epithelial dysplasia—a crucial indicator for evaluating the progression risk of precancers. Examples include squamous epithelial-derived dysplasia, such as oral leukoplakia (OLK), the precancerous disease of oral squamous cell carcinomas (OSCC),^58^ and CIN, the representative precancer of cervical carcinomas.^59,60^ In contrast, atypical adenomatous hyperplasia (AAH) exemplifies glandular dysplasia, which is predominantly observed in pulmonary and prostatic tissues during the precancerous phase of adenocarcinoma. AAH is histologically characterized by focal abnormal proliferation of atypical glandular epithelial cells.^61^ Clinically, lesions are stratified as high-risk precancers when they demonstrate either diffuse cytologic atypia or high-grade architectural disorganization.

The terminology surrounding precancerous lesions with cytologic atypia remains fraught with ambiguities and overlapping definitions. For example, distinguishing some precancers from benign neoplasms is difficult because of their shared histopathological features. This nosological overlap is exemplified by the latest 5th edition of the WHO Classification of Digestive System Tumors (2019), where benign epithelial tumors and precursors constitute a category. Moreover, in certain anatomical sites, the terminology for precancerous lesions prefers “intraepithelial neoplasia” over “epithelial dysplasia”. However, epithelial dysplasia—encompassing both cytological atypia (e.g., nuclear hyperchromasia, irregular nuclear contours) and architectural disturbances (e.g., loss of polarity, glandular crowding)—may provide a more foundational and inclusive paradigm for standardized pathological descriptions across diverse tissue types.

Unlike solid tumors, the premalignant state of hematological malignancies is challenging to identify because of the absence of distinct morphological features or discrete anatomical boundaries. Current detection strategies rely primarily on molecular diagnostics. For example, clonal hematopoiesis of indeterminate potential (CHIP), a strong risk factor for hematologic cancers,^5^ can be identified through sensitive DNA sequencing techniques. These hematological precancers represent unique models for observing stepwise natural changes from precancerous states to overt malignancy, as their natural evolution remains uninterrupted by surgical interventions or biopsy-driven perturbations.^62^ This molecular feature provides opportunities for establishing unified diagnostic criteria applicable to both hematological and solid tumor precancers.

#### Before cellular atypia

Preceding the emergence of cytologic atypia, certain persistent reactive alterations triggered by chronic adverse stimuli are also classified within the precancerous spectrum. These adaptive changes maintain normal cellular morphology without evidence of cytologic atypia.

Reactive epithelial changes primarily manifest as hyperplasia and metaplasia. In this context, hyperplasia denotes nonneoplastic cellular proliferation that rarely induces functional impairment.^63^ Metaplasia, defined as the pathological replacement of one differentiated cell type with another within the same tissue, is subclassified into three principal forms: squamous metaplasia, intestinal metaplasia (IM), and acinar–ductal metaplasia (ADM).^64^ Squamous metaplasia, observed in squamous cell carcinoma precursors, involves the substitution of native single-layered epithelia with stratified squamous epithelia. This process is frequently identified in the gastrointestinal tract and pulmonary system (e.g., tracheobronchial epithelium). Intestinal metaplasia (IM) exemplifies lineage reprogramming from native squamous or glandular epithelia to intestinal-type columnar cells. This alteration is pathognomonic in adenocarcinoma precursors of the digestive tract, notably, Barrett’s esophagus, which is the only known precursor to esophageal adenocarcinoma,^65^ and the gastric IM, a critical step in the Correa cascade preceding gastric adenocarcinoma.^66^ Acinar–ductal metaplasia (ADM), a hallmark of pancreatic ductal adenocarcinoma pathogenesis, represents the dedifferentiation of enzyme-producing acinar cells into ductal-like progenitor cells.^67^

Certain subtypes of PME, particularly stromal fibrosis resulting from persistent inflammatory responses and chronic pathogenic stimuli, are also recognized as histopathological manifestations of precancers. Substantial epidemiological evidence has established distinct correlations between pulmonary fibrosis and lung cancer, hepatic cirrhosis and hepatocellular carcinoma, and oral submucous fibrosis and OSCC development.^68,69^ This precancerous pathological process involves a dysregulated tissue remodeling response to injury in critical organ systems, including the pulmonary, hepatic, and oral mucosal tissues.^70^

Current evidence establishes that precancers represent complex systems encompassing not only cellular premalignancy but also dynamic microenvironmental modifications. Crucially, the acquisition of cellular atypia constitutes an indispensable biological checkpoint in malignant progression, whereas preatypia phases demonstrate marked pathobiological diversity. This diversity is exemplified through distinct carcinogenic trajectories: gastric carcinogenesis follows the classical Correa cascade (chronic gastritis-atrophy-intestinal metaplasia-dysplasia-adenocarcinoma),^66^ whereas oropharyngeal and cervical epithelial malignant transformation progresses through hyperplasia-dysplasia-carcinoma sequences.^58–60^ In general, precancerous states are likely attributed to an imbalance between macroenvironmental drivers, genetic and epigenetic transitions in the epithelium, and microenvironmental remodeling.

### Macroenvironmental drivers

The precancerous macroenvironment—encompassing aging, microbiome dysbiosis, obesity, and metabolic reprogramming—operates as a dynamic ecosystem that drives precancer malignancy.

#### Aging

Age-related alterations have been recognized as one of the precancerous hallmarks spanning various tissue types.^1^ Chronological aging, which is determined by the date of birth, is the primary risk factor for precancer risk, as the cumulative clinical data suggest oral,^71,72^ gastric,^73,74^ and endometrial.^75^ As reported, individuals aged over 70 years exhibit a 9-fold increased likelihood of developing gastric precancerous lesions (GPLs).^76^ Emerging evidence reveals complex dual roles of aging in tumorigenesis.

The promotion of cancer occurrence may be attributed mainly to aging-induced genomic instability.^77^ Over 50% of oncogenic mutations emerge before the onset of neoplasia.^78^ Aging has been identified as a major factor of accumulated mutations in precancers.^79^ Specifically, mutational signatures dominated by C > T substitutions at NpCpG trinucleotides demonstrate strong age-dependent correlations across both pediatric and adult malignancies, a phenomenon likely attributable to the age-associated acceleration of 5-methylcytosine spontaneous deamination.^80^ Furthermore, age-related decreases in DNA repair capacity may exacerbate genomic instability and predispose individuals to cancer. For example, mammary epithelial cells from carriers of pathogenic germline mutations in *BRCA1*, *BRCA2*, or *PALB2*, which predisposes them to breast cancer due to deficiencies in DNA damage repair, exhibit signs of accelerated aging.^81^ This premature senescence phenotype, which represents a biologically accelerated aging process, has been extensively observed across multiple precancerous states.^82,83^ Importantly, preclinical interventions targeting mucosal senescence have demonstrated efficacy in arresting precancerous lesion progression,^82,84^ thereby establishing aging as both a biomarker and a mechanistic contributor to cancer initiation.

In contrast, senescence-associated cell cycle arrest mediated by p53 or p16 activation represents an intrinsic tumor-suppressive mechanism that effectively counteracts cancer cell immortalization and restricts malignant phenotypic plasticity.^85^ For example, mice with inactivating *TP53* mutations, the most prevalent genetic alteration across human malignancies, simultaneously develop premature aging features and exhibit enhanced cancer resistance.^86^ However, malignant cells are able to escape senescence through atypical mitotic reprogramming, as evidenced by keratinocytes employing asymmetric cell division via membrane budding,^87,88^ bypassing growth arrest and promoting epithelial transformation.

The double-edged sword effect of aging on precancerous malignant transformation is also evident in its unique senescence-associated secretory phenotype (SASP).^89^ The aging process is accompanied by synergistic cellular changes in the epithelium and the microenvironment. Senescent cells have been characterized in GPLs^84^ and *Kras*-driven mouse models of pancreatic intraepithelial neoplasia (PanIN).^82^ The SASP produced by senescent cells can recruit and activate immune cells to eliminate senescent cells, restoring tissue homeostasis and preventing tumorigenesis.^90^ However, increased mutation burden and copy number changes in stromal cells occur as a function of age.^91^ Immune system function declines as organisms age, losing the ability to eliminate senescent cells within the precancerous microenvironment.^92^ The long-term persistence of senescent cells continuously secretes TGF-β, TNF-α, IFN-γ, and other factors^93,94^ creating a characteristic proinflammatory environment known as inflammaging, which promotes the development and progression of precancerous lesions.^92^ Merged data have revealed that NF-κB and JAK/STAT signaling are the major signaling pathways that stimulate the development of the SASP.^95,96^ Overall, the pathways implicated in aging and cancer prevention include p53, NF-κB, JAK/STAT, and PI3K/mTOR, among others.

#### Microbiome

Current clinical microbiome profiling reveals a common dysbiotic pattern during precancerous progression: progressive microbial diversity depletion coincides with the selective expansion of cancer-associated microbes and their genotoxic metabolites across multistage carcinogenesis.^97–100^ Pathobiont-mediated carcinogenesis occurs via host‒microbe coevolution in genetically predisposed hosts.

There are three models illustrating distinct oncogenic mechanisms, including human papillomavirus (HPV) in CINs,^34,35^ *Helicobacter pylori* (*H. pylori*) in GPLs,^101–103^ and *Candida albicans* (*C. albicans*) in OLK.^104–106^ As the first confirmed microbial driver of precancer, HPV orchestrates cervical carcinogenesis through the genomic integration of the E6/E7 oncogenes.^107^ These viral factors induce homologous recombination deficiency via E7-mediated double-strand break repair defects^108^ and activate E2F transcription factors,^109^ generating unique transcriptional responses. Host genetic modifiers (FNDC3B, BPGM) also potentiate HPV-driven epithelial transformation.^110^ *H. pylori* infection-induced gastric carcinogenesis is accelerated through host gene CD44 activation, which drive mucosal metaplasia,^111^ and by delocalizing gastric corpus-specific proteins, which destabilizes gastric homeostasis.^112^ This spatial disruption aligns with the pathogen’s tropism for the lower third of the stomach, where microbial diversity shifts corresponding to GC tumor localization.^113^ Fungal pathogenicity in OLK involves microenvironmental remodeling, including impaired fibroblastic CX3CL1 secretion,^114^ and activation of the IL-17A/IL-17RA-macrophage pathway.^115,116^

Further models reveal broader precancer–biotic interactions. Experimental models have demonstrated that repeated *Porphyromonas gingivalis* administration induces pancreatic ADM and accelerates *KRAS*-driven PanIN progression.^117^ These findings mirror the results from clinical epidemiology studies showing the associations between *P. gingivalis* oral colonization and pancreatic carcinogenesis.^117^ Microbial oncogenicity arises through direct genomic destabilization - *Escherichia coli* colonization exacerbates chemical-induced prostatic precancerous lesions,^118^ with its genotoxin colibactin inducing DNA double-strand breaks that promote TMPRSS2:ERG oncogenic fusions.^119^ Simultaneously, microbes establish preneoplastic epigenetic fields, as evidenced by hepatitis C virus-induced DNA methylation at the FOXA1/FOXA2/HNF4A transcription factors in preneoplastic liver tissue.^120^ These observations establish microbial dysbiosis as a hallmark of precancer, involving reciprocal genomic, epigenetic, and microenvironmental reprogramming.

#### Obesity

Obesity manifests as a systemic precancerous risk amplifier across multiple organ systems. Abdominal obesity and pancreatic fatty infiltration are established risk factors for Barrett’s esophagus and PanIN, respectively.^121,122^ Epidemiological studies highlight obesity-associated risks in gynecologic precancers: obese women are at increased risk of endometrial hyperplasia or polyps,^123^ cervical precancer progression with reduced screening efficacy,^124,125^ and increased recurrence postprecancer excision.^126^ Transgenerational carcinogenic effects are evidenced by maternal obesity-induced prostate hyperplasia in offspring murine models.^127^ Mechanistically, obesity orchestrates a protumorigenic macroenvironment through synergistic pathways, such as chronic inflammation, hyperinsulinemia, growth factor axis activation and mitochondrial dysfunction.^128–130^

#### Metabolism

Metabolic plasticity constitutes an essential precancerous hallmark enabling energy adaptation across oral,^131^ pulmonary,^132^ and hepatic precancers.^133^ For example, the progression of squamous metaplasia to dysplasia is fueled by a metabolic shift from glycolytic dominance to fatty acid β-oxidation,^134^ reflecting dynamic redox balance during epithelial reprogramming. *KRAS*-driven mitochondrial metabolic rewiring increases reactive oxygen species (ROS) generation,^135^ triggering acinar cell dedifferentiation into duct-like progenitors and PanIN initiation. Scholars reported a switch to aerobic glycolysis, increased c-MYC signaling, and increased serine metabolism, collectively driving the ADM process.^136,137^

Among these metabolic rewiring events, the Warburg effect, characterized by aerobic glycolysis dominance over oxidative phosphorylation,^138^ serves as a metabolic linchpin in premalignant transformation. This reprogramming enables rapid biomass synthesis in precancers, as evidenced by upregulated pyruvate levels and pyruvate kinase M2 (PKM2) isoform overexpression.^139^ In addition to supplying essential substrates, the Warburg effect promotes uncontrolled proliferation through various mechanisms. The Warburg effect may be a mutation-independent bypass of the p53 checkpoint through the STAT3/PKM2 axis or through an antioxidant defense. In precancerous local tissues, the Warburg effect can be promoted by STAT3-activated phosphorylation of PKM2.^133^ Oncogenic mutations leverage Warburg-driven NADPH overproduction to neutralize oxidative stress, as evidenced by Nrf2-induced keratinocyte survival under stress conditions.^140^ Furthermore, hyperglycemic conditions initiate a feedforward loop in which glucose-induced posttranslational modifications increase glycolytic flux, driving CRL4^COP1^-mediated p53 degradation to bypass tumor suppression.^141^ While early studies focused on transformed epithelia, emerging evidence reveals stromal metabolic symbiosis. Cancer-associated fibroblasts (CAFs) exhibit elevated PKM2/PFKFB3 expression,^142,143^ fueling tumor growth via epithelial‒mesenchymal crosstalk. However, therapeutic translation remains challenging due to drug toxicity and the suboptimal clinical efficacy of current approaches targeting the Warburg effect.^144,145^ Further elucidating the new mechanisms driving metabolic reprogramming in precancerous lesions holds transformative potential for developing targeted interventions to arrest disease progression.

### Epithelial transitions

#### Genetics in cancer predisposition

A common feature of precancers is their multifocal origins arising from the polyclonal expansion of normal progenitor cells, which undergo progressive clonal selection culminating in monoclonal dominance during malignant transformation. This paradigm has been validated across multiple tissue systems, including cervical,^146^ pancreatic,^147^ colonic,^148^ and colorectal precancers.^149,150^ The genomic aberration-based biomarkers primarily focus on somatic mutations and chromosomal instability signatures (Table 1), and the genetic landscapes shared between precancers and cancers reveal conserved oncogenic trajectories.

**Table 1:** Common precancerous biomarkers

Type	Biomarker	Function	Refs
*Oral (Oral potentially malignant disorders)*			
Genome	*TP53* (mutation); p53 (diffuse or null expression type)	Identifying high-risk patients	^412,413^
Epigenetics	5-hmC (↓)	Identifying high-risk patients	^414^
Epigenetics	H3K9ac (↑)	Associated with the early development of oral epithelial dysplasia	^415^
Transcriptome	LINC00974 (↑), TGF-β (↑)	Identifying high-risk patients	^416^
Proteome	CK13 (↓), S100A7 (↑), Decoy Receptor 2 (↑), cytoplasmic p-Smad3(↑)	Identifying high-risk patients	^216,417–419^
Proteome (blood)	CEA, SCC-Ag, ferritin (↑)	Screening biomarkers for precancers	^420^
cfDNA (blood)	*IL-6* (rs1800796 and rs1800795); pri-mir-26a-1 (rs7372209)	Genetic susceptibility to oral precancers	^421,422^
Transcriptome (saliva)	miR-10b-5p (↑), miR-182-5p (↑), miR-215-5p (↑), miR-3614-5p (↑)	Identifying high-risk patients	^423^
Transcriptome (saliva)	miR-146a (↑), miR-155 (↑)	Diagnostic biomarker for OLP	^424^
Proteome (saliva)	Paxillin (↑); Mucin1 (↑); MMP-12 (↑); LDH (↑); IL-8 (↑)	Diagnostic biomarkers for OPMDs and OSCC	^425–429^
*Cervix (Cervical intraepithelial neoplasia)*			
Microbiome	HPV, HIV, *Gardnerella vaginalis*, and *Trichomonas vaginalis*	Risk factors for cervical precancers	^430,431,432^
Microbiome	HPV16, 18, 31, 33, 58	Risk factors for cervical precancers	^433–435^
Microbiome	HPV16/18 E6 oncoprotein	Screening biomarkers	^436^
Microbiome	Higher methylation at specific *E1* CpGs, HPV-16 E6/E7 genetic variations (D32E/M28V/L94P)	Associated with an increased risk	^437,438^
Genome	*PAX8* (rs10175462), *CLPTM1L* (rs27069)*, HLA-DQA1* (rs9272050)	Genetic susceptibility to cervical cancer	^439^
Epigenetics	DNA methylation at cg13944175 (*ATP10A*) and cg03419058 (*HAS1*)	Identifying high-risk patients	^440^
Proteome	CK17 (↑), TWIST (↑), SNAIL (↑), SLUG (↑)	Identifying high-risk patients	^441,442^
Transcriptome (blood)	miR-25 (↑), KLF4 (↓)	Associated with cervical cancer development	^443^
*Stomach (Gastric precancerous lesions)*			
Microbiome	*H. pylori*		^383,444^
Genome	*PSCA* (rs2920283), *IL-21* (rs907715, rs12508721, rs2221903 and rs907715)	Genetic susceptibility to gastric atrophy	^444,445^
Genome	Chr 7, 8 (segments amplification)	Diagnostic biomarker of GPLs	^446^
Epigenetics	NOS2 (↑), DNA methylation (↑)	Associated with intestinal metaplasia	^447^
Transcriptome	Cytoplasmic Drosha (↓)	Prognostic biomarker	^448^
Transcriptome	miR-21 (↑), miR-142 (↓), miR-223 (↓)	Biomarker of gastric precancers	^449^
Transcriptome (blood)	EV-derived GClnc1, miR-22-3p (↑)	Biomarker of gastric precancers	^450,451^
Proteome	EGF (↑)	Associated with atrophic gastritis	^217^
Proteome (blood)	IL-4 (↑), IL-5 (↑), Omp (↑), HP0305 (↑), pepsinogen II and anti-*H. pylori* IgG combined, CagA (+), GroEL (+)	Identifying high-risk patients	^452,453–455^
Urine	ANXA11, CDC42, NAPA and SLC25A4 (↑)	Identifying high-risk patients	^456^
*Esophagus (Barrett esophagus)*			
Blood	NLR	Identifying high-risk patients	^457^
Microbiome (saliva)	*A.g; P.g; H.h; S.a; F.n*	Diagnostic biomarkers for precancer and cancer	^458^
Genome	*EGF* (rs4444903)	Genetic susceptibility to Barrett’s esophagus	^459^
Genome	TP53 biallelic inactivation	Identifying high-risk patients	^228^
Proteome	TAGLN2 (↑), CRNN (↓)	Risk biomarkers for ESCC	^460^
Proteome	Claudin-2 (↑), p53 (↑), CEA (↑), CA19-9 (↑), NAPRT (↑), PLCE1 (↑), IKBα (↑)	Identifying high-risk patients	^461–464^
Transcriptome	hTERC (amplification)	Diagnostic and prognostic biomarkers for precancer	^465^
Colorectum			
Blood	Treg (↑), Tfh cell(↓)	Identifying high-risk patients	^466^
Genome	PIK3CA (amplification)	Identifying high-risk patients	^247^
Transcriptome	miR-1-3p (↓), CDK6 (↑)	Diagnostic biomarkers for precancer	^467^
Epigenetics (blood)	SEPT9, SDC2, ALX4 (hypermethylation)	Identifying high-risk patients	^468^
Proteome	CK7 (↑), TP53 (↑), ATF6 (↑)	Identifying high-risk patients	^469,470^
Proteome (blood)	FGF-21 (↑)	Identifying high-risk patients	^471^
Metabolome (urine)	PGE_2_ (PGE-M)	Identifying high-risk patients	^472^
*Liver*			
Transcriptome	IFN-I/miR-484 (↑)	Identifying high-risk patients	^215^
Proteome (blood)	KR1B1 (↑)	Identifying high-risk patients of MASLD	^473^
Proteome (blood)	LDHA in T cells (↑)	Identifying high-risk patients of NAFLD	^474^
Proteome	UBE2D1 ( ↑ ), IL-6 (↑)	Identifying high-risk patients	^232^
Proteome	PKM2 and p-STAT3 (↑)	Biomarkers of liver precancerous lesions	^133^
*Gallbladder*			
Genome	Telomere length (↓)	Identifying high-risk patients	^475^
*Laryngeal*			
Blood	NLR, PLR, MLR	Diagnostic biomarkers for vocal fold leukoplakia	^476^
Genome	PIK3CA and SOX2 (amplification)	Identifying high-risk patients	^246^
*Pancreatic (Pancreatic intraepithelial neoplasia)*			
Epigenetics	5-hmC (↓)	An early event in pancreatic tumorigenesis	^477^
Epigenetics	Reprogramming-mediated repression of somatic cell enhancers with *Kras* mutation	Identifying high-risk patients	^478^
Proteome	CRABP-II (↑)	Identifying high-risk patients	^479^
Proteome	PYK2 (↑)	Associated with acinar-to-ductal metaplasia	^236^
Proteome (blood)	apoA2-I (↓)	Identifying high-risk patients	^480^
*Skin (Actinic keratosis)*			
Proteome	MCPIP1 (↓), TGF-β (↑)	Identifying high-risk patients	^221^
Proteome	Ki67 (↑), p16 (↑), cyclin A (↑), membranous β-catenin (↓)	Identifying high-risk patients	^481,482^

*5-hmC* 5-hydroxymethylcytosine, *8-OHdG* 8-hydroxy-2-deoxyguanosine, *apoA2-I* apolipoprotein A2-isoforms, ATF6-activating transcription factor 6, *A.g*. Actinomyces graevenitzii, *ATP10A* ATPase phospholipid transporting 10A, *BE* Barrett’s esophagus, *CRABP-II* cellular retinoic acid-binding protein II, *CK* cytokeratin, *CIN* cervical intraepithelial neoplasia, *CEA* carcinoembryonic antigen, *cfDNA* cell-free DNA, *EAC* esophageal adenocarcinoma, *EGF* epidermal growth factor, *EGFR* epidermal growth factor receptor, *ESCC* esophageal squamous cell carcinoma, *EV* extracellular vesicle, *F.n*. Fusobacterium nucleatum, *FGF-21* fibroblast growth factor 21, *GPLs* gastric precancerous lesions, *GC* gastric cancer, *HAS1* hyaluronan synthase 1, *HCC* hepatocellular carcinoma, *hTERC* human telomerase RNA component, *H.h*. Haemophilus hemolyticus, *H. pylori Helicobacter pylori*, *HPV* human papillomavirus, *HIV* human immunodeficiency virus, *IL* interleukin, *IFN* interferon, *LDHA* lactate dehydrogenase A, *LDH* lactate dehydrogenase, *MCPIP1* monocyte chemotactic protein 1-induced protein 1, *MTHFR* methylenetetrahydrofolate reductase, *MDA* malondialdehyde, *MMP* matrix metalloproteinases, *MASLD* metabolic dysfunction-associated steatotic liver disease, *miRNA* microRNA, *MLR* monocyte-to-lymphocyte ratio, *NAPRT* nicotinamide phosphoribosyltransferase, *NAFLD* nonalcoholic fatty liver disease, *NLR* neutrophil-to-lymphocyte ratio, *OSCC* oral squamous cell carcinoma, *OLP* oral lichen planus, *P.g Porphyromonas gingivalis*, *PCDH10* protocadherin 10, *PCDH17* protocadherin 17, *PIK3CA* phosphatidylinositol-4,5-bisphosphate 3-kinase catalytic subunit alpha, *PKM2* pyruvate kinase M2, *PLCE1* phospholipase C epsilon-1, *PYK2* proline-rich tyrosine kinase 2, *PLR* platelet-to-lymphocyte ratio, *PGE*_*2*_ prostaglandin E2, *S100A7* S100 calcium binding protein A7, *SCC-Ag* squamous cell carcinoma antigen, *S.a* Streptococcus australis, *TAGLN2* transgelin-2, *TLR* toll-like receptor, *Tfh cell* T follicular helper cell, *Treg* regulatory T cell, *VEGF* vascular endothelial growth factor

The molecular pathogenesis of precancerous states is hierarchically organized: initial driver mutations dysregulating cell cycle progression establish the premalignant niche, whereas subsequent hits in apoptosis evasion, differentiation blockade, and metabolic–immune reprogramming orchestrate the transition to invasive malignancy.^151^ Sessile serrated adenomas/polyps, established colon precancers, are initiated by *BRAF* mutations with a methylation phenotype.^152^ Breast precancers demonstrate somatic truncating mutations affecting the prolactin receptor,^153^ whereas *KRAS* variants represent ubiquitous hotspot mutations shared by precancerous lesions and tumors.^147,154,155^ The *MUC2* variants inversely correlate with gastric metaplasia regression,^156^ and *BRA*F-altered AAH (23% prevalence) frequently coexists with *EGFR*-mutant adenocarcinomas.^157^ Especially with the accumulation of large-scale population genomic data, hotspot mutations in signaling pathways such as p53, PI3K, TGF-β, and Wnt are also observed in precancerous diseases. They will be discussed in detail in the relevant sections dedicated to these pathways.

Chromosomal instability, a priming event in tumorigenesis, may emerge even earlier than many driver mutations, as evidenced by premalignant lung and Barrett’s esophagus data.^158,159^ By inducing large-scale genomic imbalances, chromosomal instability drives the accumulation of copy number variations (CNVs), particularly at loci harboring oncogenes and tumor suppressor genes. For example, paired esophageal SCCs and intraepithelial neoplasia exhibit overlapping CNVs in genomic regions governing DNA repair, apoptosis, proliferation, and cell adhesion, notably at 11q13.3 (*CCND1*), 3q26.33 (*SOX2*), 2q31.2 (*NFE2L2*), and 9p21.3 (*CDKN2A*).^160^ Hepatocarcinogenesis models reveal an initial loss of DNA copy numbers of tumor suppressor genes in the 4q and 13q regions,^161^ thereby conferring survival benefits to cancer cells. Genomic analyses of Barrett’s esophagus progression identified 9p21.3 CNVs (encompassing *FHIT* exon 5 and *CDKN2A/B* loci) as cardinal genomic events in 88% of esophageal adenocarcinoma cases,^162^ while 9p13 amplifications that activate oncogenes, such as *VCP, DCTN3*, and *STOML2*, drive phenotypic diversification in oral precancer-to-cancer transitions.^163^

#### Epigenetic factors related to cancer risk

A recent *cell* study revealed that transient disruption of Polycomb-mediated transcriptional silencing can epigenetically reprogram cell fate, inducing malignant transformation in *Drosophila* even without oncogenic driver mutations.^164^ This evidence strongly underscores epigenetic dysregulation as an essential tumor-initiating mechanism. Epigenetic modifications—including DNA methylation (DNAme), histone posttranslational modifications, and noncoding RNA-mediated regulation—orchestrate gene expression patterns that define cancer predisposition.

DNAme aberrations have emerged as one of the earliest indicators of epigenetic changes present in precancers, including hepatitis virus-associated chronic liver injury,^165^ gastric IM,^166^ and other precancers, affecting regulatory hubs such as enhancers,^120^ gene bodies,^167^ promoters,^168^ CpG islands, and DNA-binding regions of transcription factors.^169^ Mechanistically, genomic DNAme is orchestrated by the DNA methyltransferase (DNMT) family and the DNA demethylase (TET) family. The overexpression of this DNAme “guardian” DNMT1 paradoxically drives cancer-specific hypomethylation during oral carcinogenesis.^170^ Furthermore, TET1 oxidizes 5-methylcytosine (5mC) to 5-hydroxymethylcytosine (5hmC), activating DNA hypomethylation. In cervical carcinogenesis, escalating TET1/5hmC levels correlate with lesion progression, peaking in high-grade squamous intraepithelial lesions. TET1-mediated 5hmC enrichment at *ZEB1* and *VIM* promoters drives epithelial‒mesenchymal transition (EMT) by erasing H3K4me3 (activating mark) and recruiting H3K27me3 (repressive mark).^171^ In addition to the DNAme, histone modification dynamics critically shape precancerous plasticity. The H3K27me2/3 demethylase KDM6B is upregulated in PanINs, and KDM6B loss exacerbates cellular aggressiveness.^172^ Pharmacological modulation of DNMT, TET, or histone demethylases may disrupt the epigenetic priming of malignant fate in precancers.

### Microenvironmental modulation

The precancer microenvironment (PME) is a dynamically evolving microcosmic ecosystem that envelops precancerous lesions and early-stage tumors. PMEs include stromal cells (e.g., activated fibroblasts), immune infiltrates (e.g., regulatory T cells and M1/M2 macrophages), vasculature networks, and noncellular elements such as remodeled extracellular matrix (ECM), hypoxia gradients, and soluble signaling molecules (e.g., cytokines and growth factors). Mirroring features of the tumor microenvironment (TME),^173^ the PME actively shapes lesion heterogeneity and steers malignant progression through bidirectional crosstalk between premalignant cells and their surroundings.

#### Extracellular matrix remodeling

Dysregulated deposition of ECM components serves as a cornerstone of pathological tissue remodeling, driving fibrosis progression through biomechanical and biochemical perturbations.^174^ ECM remodeling dynamically modulates cellular signaling cascades, with fibroblast activation and phenotypic transition emerging as central mediators of stromal ECM deposition.^175^ Stromal desmoplasia, which is characterized by pathological collagen deposition and expansion of activated fibroblast populations, is a distinguishing feature separating precancerous lesions from invasive carcinomas across multiple malignancies, including breast, esophageal, gastric, and oral precancer.^176–179^ Oral submucous fibrosis (OSF), an arecoline-induced oral precancer, exemplifies stromal deposition as a carcinogenic catalyst. Key pathogenic drivers may include transcriptional reprogramming and prosurvival signaling. Twist1-mediated EMT and HOTTIP lncRNA overexpression amplify collagen gel contraction and fibroblast motility,^179,180^ and EGFR/ERK hyperactivation sustains fibrogenic phenotypes through autocrine loops.^181^ Moreover, single-cell transcriptomic profiling has revealed functionally polarized fibroblast subpopulations within premalignant niches, classified as matrix-remodeling, inflammatory, vascular-associated, and antigen-presenting fibroblasts. The fibroblast subpopulations, characterized by the expression of extracellular matrix components (POSTN, COL6A1, and COL6A2) or markers of contractile fibroblasts (ACTA2, MYL9, and ACTG2), are present in the precancerous stages.^177^ As detailed in our previous review, these heterotypic fibroblast states collectively permit malignant transformation through biomechanical and biochemical crosstalk.^182^

#### Immune dysregulation

The transition from precancerous lesions to invasive cancers is marked by dynamic immune reprogramming,^183^ characterized by the progressive attenuation of antitumor immunity (e.g., cytotoxic T-cell depletion^184^), the concomitant expansion of immunosuppressive networks (e.g., immunosuppressive myeloid and stromal subsets^178^) and a concomitant increase in inflammation.^184^ This immune-editing process fosters an immunosuppressive microenvironment in which malignant clones evade surveillance. For example, progressing lung premalignancies are enriched in immunosuppressive gene signatures (e.g., IL12A and GZMB), which are correlated with impaired T-cell effector function.^157^ Compared with GPLs, early gastric cancers exhibit increased immune activity (e.g., CD8 + T-cell and macrophage infiltration),^185^ suggesting an unsuccessful immune response before full immunosuppression. Gas6/Axl-driven survival signaling in macrophages promotes epithelial disorganization via Akt/STAT3-mediated E-cadherin dysregulation.^186^ Epigenetically, global DNA hypomethylation in precancers is associated with “immune-cold” phenotypes;^187^ 9p21.3 loss of heterozygosity in oral potentially malignant disorders cooccurs with epithelial PD-L1 expression and CD8 + T-cell exhaustion;^188^ mutations, CNVs, and DNAme aberrations may collectively facilitate immune evasion, enabling fit precancerous subclones to dominate during malignant transformation.^183^ Given the convenience of blood-based tests,^132^ the circulating immune, genetic, and epigenetic signatures (e.g., PD-L1+ exosomes, ctDNA, and cfDNA methylation) offers a minimally invasive strategy for early detection.

#### Inflammation

Inflammation-driven oncogenesis is a pathway of precancerous malignant transformation that operates through context-dependent mechanisms that balance tumor-suppressive clearance and mutagenic promotion.^151,189^ While controlled inflammation eliminates DNA-damaged cells via apoptosis (e.g., the caspase-8-mediated DNA damage response), chronic inflammation fosters genomic instability by overwhelming the DNA repair capacity and selecting for prosurvival mutations.^151^ For example, progressive caspase-8 inactivation and mutational accrual occur during the OLK-to-carcinoma transition, exacerbated by inflammation-induced immune surveillance collapse.^184^ In gastric premalignancy, *NKX6.3* deficiency triggers gastric mucosal atrophy via the upregulation of inflammatory cytokines,^190^ linking transcriptional dysregulation to inflammatory tissue remodeling. IL-33-activated eosinophils lead to intestinal metaplasia in gastritis-prone mice, mechanistically bridging the inflammation‒metaplasia‒dysplasia axis through STAT5-dependent epithelial reprogramming.^191^ Chemokines—a multifunctional family of chemotactic cytokines—orchestrate PME formation through immune cell recruitment and stromal crosstalk.^192^ Clinically exploitable features include salivary chemokine profiling^193^ and prognostic models.^194^ The key signaling hubs linking inflammation to malignant transformation include the NF-κB, JAK/STAT, and TGF-β signaling pathways.

#### Angiogenesis

The uncontrolled proliferation of tumor cells is physiologically constrained by the diffusion limits of oxygen and nutrients, necessitating the activation of the “angiogenic switch” to breach the 0.2 mm capillary diffusion barrier.^195,196^ This switch—orchestrated by proangiogenic signaling cascades—is frequently triggered in precancerous lesions, enabling nascent clones to establish vascular networks for sustained growth.^197^ Dysplasia and carcinoma in situ exhibit subepithelial microvascular irregularities and neoangiogenesis. VEGF-A expression increases during the OLK-to-carcinoma transition, with angiogenic activity paralleling the histological grade.^198^ In Barrett’s esophagus, premalignant cells secrete VEGF to activate VEGFR2-dependent PLC-PKC-ERK signaling, fostering autocrine proliferation loops and paracrine angiogenesis; owing to the accumulation of VEGF, macrophage-secreted MMP-9 degrades ECM barriers, amplifying perilesional neovascularization.^199,200^ Key signaling pathways related to precancerous angiogenesis include the Hedgehog pathway and the HIF-1α pathway.

### Soil degeneration

The malignant transformation of precancers is driven by synergistic alterations spanning genomic reprogramming and macro/microenvironmental remodeling. Key hallmarks include aging-associated dyshomeostasis, host‒microbiome interactions, metabolic dysregulation, genomic/epigenetic reprogramming, stromal‒immune remodeling, and angiogenic switching within the microenvironment.

**Fig. 3:**
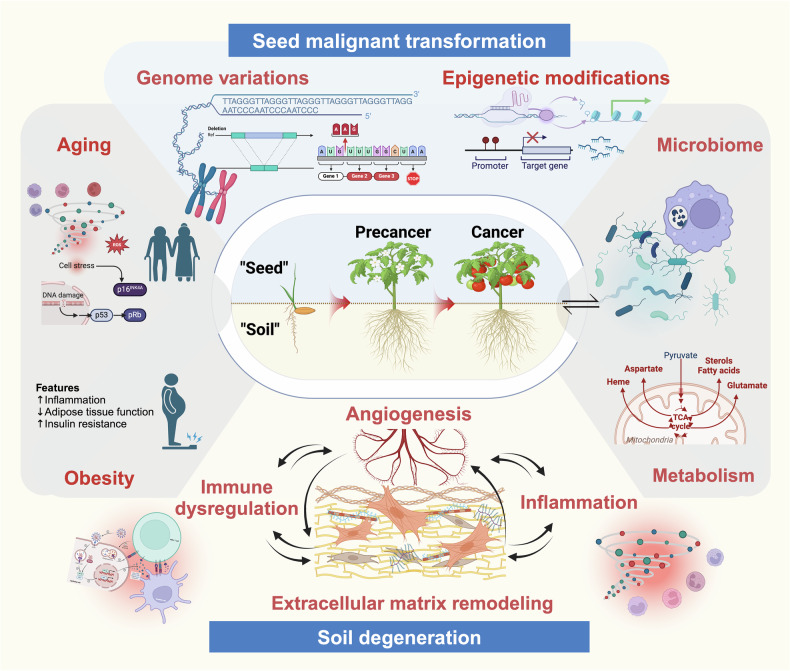
Malignant seed transformation and soil degeneration in multistep transformation processes. The malignant evolution of precancers is the combined effect of intrinsic uncontrolled cell proliferation (seed malignant transformation) and extrinsic environmental remodeling (soil degeneration). The multistep processes involve dynamic events including aging, host‒microorganism interactions, obesity, metabolic abnormalities, genome/epigenetic changes that drive epithelial malignancy, and microenvironmental reprogramming encompassing extracellular matrix remodeling, immune dysregulation, inflammatory infiltration, and pathological angiogenesis

Contemporary frameworks emphasize the gatekeeper role of the environment in maintaining premalignant lesion dormancy versus triggering autonomous proliferation. This concept builds upon Paget’s seminal “seed and soil” hypothesis,^201^ which originally described how metastatic cancer cells (“seeds”) colonize permissive distant niches (“soil”). Modern adaptations extend this metaphor to primary tumors.^202,203^ Concepts have emerged when applied to the precancerous stage, including field cancerization,^204^ the tissue microenvironment,^205^ the tumor-like microenvironment,^206^ and the PME. Together, these concepts reveal the coevolution of the microenvironment and transformed cells, indicating a “vicious” microenvironment. To unify these observations, we propose “*soil degeneration*”—a degenerative process in which cumulative microenvironmental damage (e.g., stromal deposition, chronic inflammation, metabolic reprogramming, and immune dysregulation) is induced by both intrinsic and extrinsic stressors (Fig. 3). Soil degeneration enables premalignant clones to evade growth suppression and achieve competitive fitness, ultimately driving malignant transformation.

This comprehensive framework redefines precancers by transitioning from a lesion-focused paradigm to a systemic perspective. Increasing evidence highlights the rapid responsiveness of the microenvironment to both extrinsic and intrinsic stimuli. Investigations into aging-associated mechanisms in precancers reveal a prematurely senescent microenvironment, particularly within fibroblasts, endothelial cells, and immune populations;^207^ senescent fibroblasts in the subepithelial stroma promote the malignant transformation of adjacent epithelial cells via increased TGF-β secretion.^93^ The fibroblast-estrogen signaling axis in CIN provides another compelling example. Estrogen receptor alpha (ERα)-mediated signaling in cervical carcinogenesis operates primarily through stromal fibroblasts rather than tumor cells themselves.^208^ Crucially, microenvironmental cross-talk orchestrates multiple hallmarks of precancers. For example, fibroblast-induced inflammation plays a significant role in microbiome-related malignant transformation of precancerous lesions,^114^ whereas fibroblast- and immune cell-induced angiogenesis are pivotal for precancerous progression.^209,210^ Notably, stromal modifications may even precede epithelial malignancy. A seminal study by Bhowmick et al. demonstrated that in a transgenic mouse model, TGF-β receptor II (TβRII) ablation in murine fibroblasts spontaneously induces forestomach squamous carcinoma and prostatic intraepithelial neoplasia,^211^ establishing the stroma as a potential initiator of carcinogenesis. Collectively, these findings position the PME not only as a transformation accomplice but also as a dominant instigator and precursor to malignancy.

## Major signaling pathways in precancer initiation and progression

Numerous signaling pathways play critical roles in the pathogenesis of precancers. Through systematic classification of two decades of literature on precancers (identified via abstract/keyword screening), we identified six principal mechanistic domains involving more than 10 key pathways: the TGF-β-mediated epithelial–stromal crosstalk; the p53, PI3K/mTOR, and NF-κB pathways in aging-related premalignancy; mutational drivers in the p53, Wnt, PI3K, MAPK, and EGFR pathways that govern cell identity transitions; the NF-κB, JAK/STAT, and Hippo signaling pathways that mediate responses to immune dysregulation and chronic inflammation; the STAT and HIF-1 pathways that coordinate nutrient adaptation and metabolic shifts; and Hedgehog signaling that modulates angiogenesis.

### TGF-β signaling pathway

**Fig. 4:**
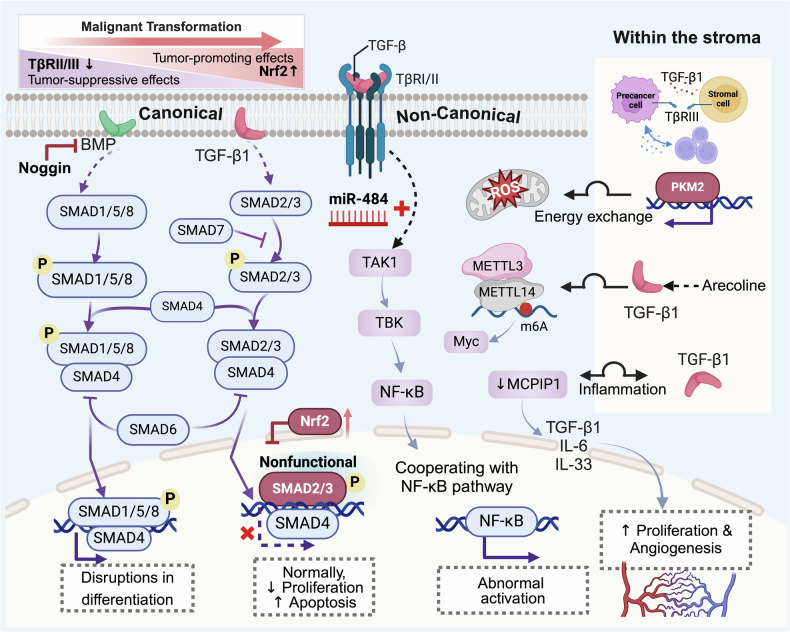
TGF-β signaling pathway in precancers. The tumor-suppressive function of the TGF-β signaling pathway is frequently lost during the development of precancerous lesions. This loss primarily results from alterations in components of the canonical SMAD pathway, which disrupt the pathway’s ability to induce cell cycle arrest and apoptosis. Interactions with other non-SMAD signaling pathways and activation within the evolving precancer microenvironment can further facilitate the progression towards malignancy. BMP bone morphogenetic protein, SMAD mothers against decapentaplegic homolog, TAK1 TGF-β-activated kinase 1, TBK TANK-binding kinase, METTL3/14 methyltransferase-like 3/14, MCPIP1 monocyte chemotactic protein 1-induced protein 1, PKM2 enzyme isoform 2 of pyruvate kinase

The canonical transforming growth factor-beta (TGF-β) signaling pathway initiates ligand binding to a heterotetrameric receptor complex comprising TGF-β type I (TβRI) and type II (TβRII) receptors. This interaction triggers TβRII-mediated phosphorylation of TβRI, which subsequently activates the downstream effector SMAD2/3 through C-terminal phosphorylation. Phosphorylated SMAD2/3 forms a trimeric complex with SMAD4, translocates to the nucleus and binds SMAD-binding elements (SBEs) to regulate the transcriptional programs governing cellular homeostasis. Notably, TGF-β signaling exhibits context-dependent duality, functioning as either a tumor suppressor or promoter. This paradoxical behavior arises from alterations in pathway components, including TβRII, SMAD2, and SMAD4 depletion/mutation.^212^ In precancerous contexts, TGF-β pathway dysregulation drives oncogenesis through three interconnected mechanisms **(**Fig. 4**)**. This is due to canonical SMAD pathway aberrations, noncanonical pathway activation, and stromal reprogramming.

The normal transmission of the canonical SMAD pathway in precancers weakens via receptor depletion and SMAD signaling interruption. Therapeutic targeting of TβRI/II or TβRIII significantly attenuates the formation and progression of precancerous lesions and demonstrates potent tumor growth suppression in vivo.^213–215^ In oral premalignancy, cytoplasmic phosphorylated SMAD3 (p-SMAD3) creates a nonfunctional state.^216^ In addition, bone morphogenetic protein (BMP) pathway inhibition disrupts gastric lineage differentiation. Physiological BMP/EGF gradients normally orchestrate parietal-chief cell differentiation along the gland axis. BMP pathway activation is concomitant with foveolar differentiation, whereas Noggin-mediated BMP blockade induces differentiation arrest.^217^

The TGF-β pathway also drives precancerous progression through dynamic crosstalk with noncanonical signaling cascades and microenvironmental reprogramming. In precancerous liver lesions, TGF-β cooperates with NF-κB via miR-484 overexpression to initiate tumorigenic reprogramming.^215^ TGF-β1 induces ERK pathway activation through a noncanonical pathway to mediate EMT.^218^ Furthermore, stromal TGF-β1 signaling plays a key role in promoting the malignant transformation of precancers through mechanisms such as metabolism, ECM remodeling, and inflammation. TβRII downregulation in fibroblasts triggers nuclear PKM2 translocation, reprogramming glucose metabolism to fuel premalignant and cancer cells.^142^ Exposure to the carcinogen arecoline activates a TGF-β1-METTL3/14 m6A methyltransferase axis in fibroblasts, driving epithelial epigenetic remodeling.^219,220^ TGF-β1 sustains a protumorigenic feedback loop of unrestrained inflammatory signaling by suppressing Mcpip1, an anti-inflammatory RNase, in premalignant keratinocytes.^221^ In *Kras*-driven lung carcinogenesis, TGF-β1 accumulation and immune cell infiltration are evident at the AAH and during adenoma outgrowth, fostering permissive niches for subpleural precancerous expansion.^222^

### p53 signaling pathway

**Fig. 5:**
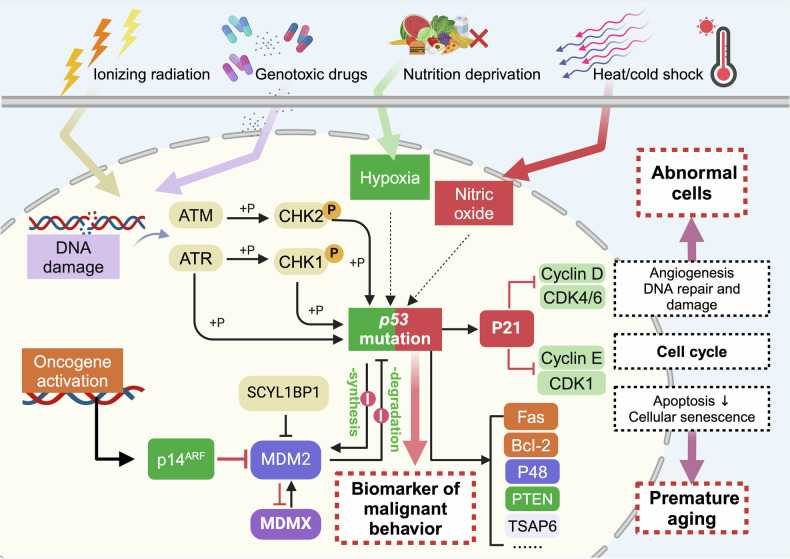
p53 signaling pathway in precancers. Wild-type p53 promotes cell cycle arrest primarily by inducing the expression of p21^Cip1^, or triggers apoptosis primarily by inducing the expression of proapoptotic proteins and repressing anti-apoptotic proteins. However, during carcinogenesis, the p53 gene is frequently mutated. Mutant p53 loses the transcriptional activity of wild-type p53 and fails to effectively induce its target genes, including MDM2. Mutant p53 proteins often undergo conformational changes that prevent their recognition and degradation by MDM2. This results in the abnormal accumulation of stable, dysfunctional mutant p53 proteins that not only lose tumor-suppressive functions but often acquire oncogenic gain-of-function, contributing to cancer progression. ATM ataxia-telangiectasia mutated proteins, ATR ataxia-telangiectasia mutated- and Rad3-related proteins, CHK1/2 checkpoint kinase 1/2, CDK cyclin-dependent kinases, Bcl-2 B-cell lymphoma-2, PTEN phosphatase and tensin homolog, TSAP6 tumor suppressor-activated pathway 6

The tumor suppressor p53 plays a dualistic role in carcinogenesis, with its functional consequences critically determined by mutational status. While wild-type (WT) p53 serves as a tetrameric transcription factor that maintains genomic stability through cell cycle arrest (via p21^Cip1^),^223^ apoptosis initiation (through Bcl-2 family members and caspase cascade activation),^224^ and senescence enhancement, its frequent mutation drives oncogenic reprogramming in almost all types of precancers.^55^ WT p53 activity is tightly regulated by MDM2-mediated ubiquitination, forming an autoregulatory loop that prevents pathological accumulation linked to premature aging.^225^ Mutant p53 proteins acquire properties via failed MDM2 induction, resulting in tumor-specific accumulation.^226^ This biological dichotomy creates diagnostic complexity, as nuclear p53 accumulation observed via immunohistochemistry paradoxically indicates either mutagenesis or WT p53 hyperactivation.^227^ Thus, while p53 functions as a genomic guardian preventing abnormal cell proliferation (Fig. 5), its mutational subversion represents a critical transition enabling tumorigenesis, necessitating a nuanced interpretation of p53 status in clinical assessments.

*TP53* mutations and functional dysregulation emerge as pivotal initiating events in malignant transformation, with biallelic inactivation frequency markedly increasing during early precancer evolution—a prerequisite for the acquisition of oncogenic CNVs in the cell cycle, DNA repair, and apoptosis regulators.^228^ This genomic destabilization enables ΔNP63-driven epithelial reprogramming via Src/Erk/AKT-mediated EMT activation and cell cycle dysregulation, progressing from basal layer-restricted abnormalities in early lesions to entire layer abnormalities in advanced malignancies.^229^ Paradoxically, certain *TP53* variants exhibit context-dependent tumor-suppressive capacities—a specific double mutation (p53^53,54^) in the TAD2 domain demonstrates enhanced PanIN suppression compared with wild-type p53 in pancreatic carcinogenesis.^230^ In addition to genetic alterations, precancerous p53 inactivation occurs through posttranslational mechanisms: lipid peroxidation-derived isolevuglandins modify p53 to impair promoter binding and acetylation-dependent transcription,^231^ whereas UBE2D1-mediated ubiquitination drives p53 degradation in HCC premalignancy.^232^ This multilayered regulation creates a status where both p53 deficiency and overexpression prove pathogenic; insufficient activity permits premalignant evolution, whereas excessive signaling accelerates aging. Consequently, p53-targeted therapies must carefully exploit mutation-specific vulnerabilities.

### Wnt signaling pathway

**Fig. 6:**
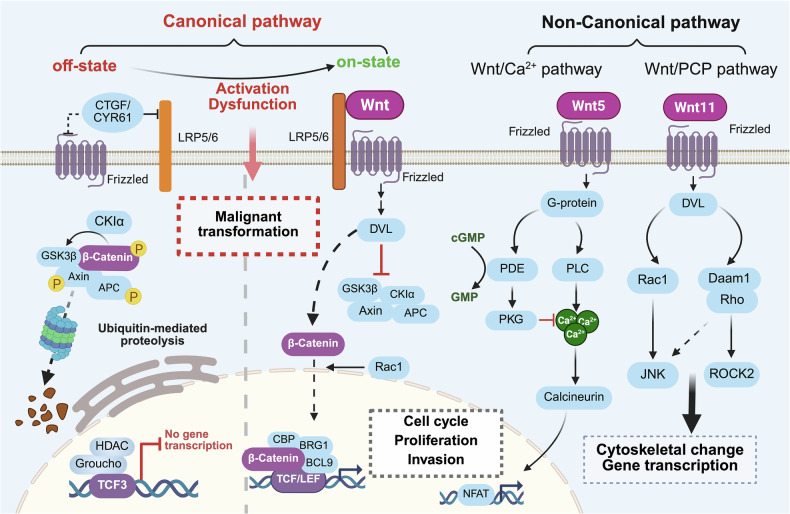
Wnt signaling pathway in precancers. The Wnt pathway is categorized into β-catenin-dependent (canonical) and β-catenin-independent (noncanonical) signaling. Precancers frequently exhibit dysregulated canonical Wnt/β-catenin signaling. The canonical pathway primarily regulates cell fate and proliferation. In contrast, noncanonical signaling comprises the Wnt/PCP pathway, regulating planar cell polarity through cytoskeletal dynamics, and the Wnt/Ca^2+^ pathway, modulating adhesion and migration. CTGF connective tissue growth factor, Cyr61 cysteine-rich 61, LRP5/6 low-density lipoprotein receptor protein 5/6, CK1α casein kinase 1α, DVL Dishevelled, GSK3β glycogen synthase kinase-3β, HDAC histone deacetylases, WLS Wntless, WIF1 Wnt inhibitory factor 1, Cer1 cerberus 1, Rac1 Rac family small GTPase 1, TCF transcription factor, LEF lymphoid enhancer binding factor, Brg1 brahma-related gene 1, NFAT nuclear factor of activated T cells, PDE phosphodiesterases, PKG protein kinase G, PLC phospholipase C, JNK c-Jun N-terminal kinases, MAPK mitogen-activated protein kinase

The evolutionarily conserved Wnt signaling pathway orchestrates developmental patterning and tissue homeostasis through β-catenin-dependent (canonical) and -independent (noncanonical) branches, with canonical Wnt/β-catenin activation driving nuclear β-catenin-TCF complex formation and target gene transcription (Fig. 6). This pathway has emerged as a linchpin in precancerous pathogenesis, as evidenced by Wnt-Kras coactivation, which induces biliary precancer,^233^ and Wnt-driven genomic instability, which propels the adenoma‒carcinoma sequence in colorectal carcinogenesis.^234^ Molecular epidemiology reveals that Wnt pathway polymorphisms (*GSK3B*/rs9879992 and *AXIN2/*rs3923087) are risk determinants for oral potentially malignant disorders (OPMDs),^235^ whereas Wnt1 mammary precancers yield adenocarcinoma from Keratin 6a-expressing precancerous stem cells and squamous metaplastic carcinoma from differentiated whey acidic protein-positive subset cells.^20^ In sessile serrated adenomas, i.e., colorectal cancer precursors, nuclear β-catenin accumulation is correlated with AXIN2/MCC promoter methylation,^234^ indicating that epigenetic dysregulation synergizes with canonical Wnt activation during early serrated carcinogenesis. These findings position Wnt signaling as a main regulator of premalignant clonal evolution, operating through genetic, epigenetic, and epithelial differentiation mechanisms that collectively drive malignant transformation.

The Wnt/β-catenin pathway also drives precancerous progression through multifaceted molecular crosstalk, serving as a nexus for the oncogenic reprogramming of precancers. Proline-rich tyrosine kinase 2 phosphorylates β-catenin at Y654, amplifying Wnt signaling to fuel mutant *Kras*-driven ADM and PanIN formation.^236^ Concurrently, cadherin switching (E-cadherin loss/N-cadherin redistribution) in the pancreatic epithelium synergizes with β-catenin nuclear translocation to orchestrate EMT.^237^ Gastric precancerous evolution involves OLFM4-MYH9 complexes that destabilize GSK3β via ubiquitination, unleashing β-catenin-mediated proliferation and invasion in GPLs.^238^ Pathogen-mediated Wnt activation further accelerates premalignant transformation, exemplified by *H. pylori*-induced β-catenin stabilization in the gastric mucosa.^239^ The Wnt inhibitor compound 1, which suppresses TCF/lymphoid enhancer-binding factor activity, decreases nuclear p-β-catenin (Y489) and induces ciliated cell differentiation.^240^ TCF transcriptional activity is also activated by ectopic expression of PDE10 in normal and precancerous colonocytes to increase cell proliferation.^241^ Depletion of Claudin18.2-mediated immune response affects the Wnt signaling pathway and PD-1 pathway.^242^ Clinically, nuclear β-catenin accumulation has emerged as a histopathological biomarker for risk prediction and therapeutic stratification, reflecting the central role of the pathway in bridging genetic alterations and microenvironmental reprogramming.

### PI3K signaling pathway

**Fig. 7:**
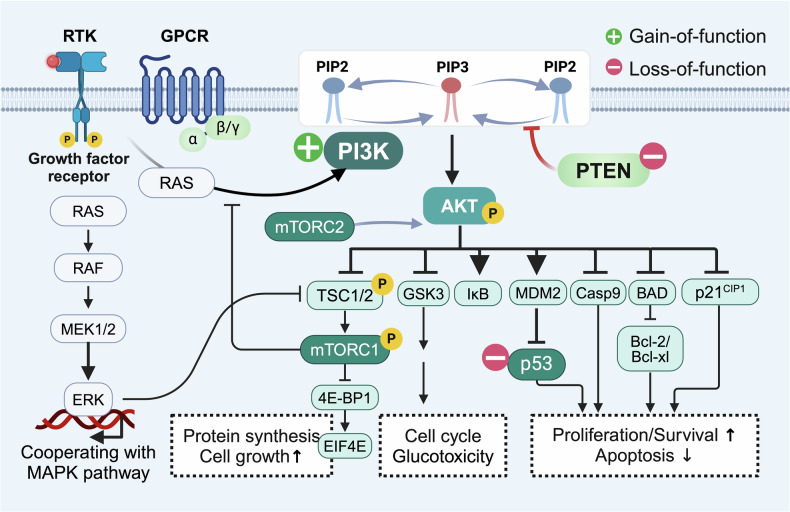
The PI3K signaling pathway in precancers. The PI3K signaling pathway is dysregulated in almost all cancers and precancerous lesions. The dysregulation of the PI3K signaling pathway in precancers is partly attributed to mutations or amplifications in PI3K catalytic subunits (e.g., PIK3CA, PIK3CB) and/or loss-of-function mutations, reduced expression, or inactivation of the tumor suppressor PTEN. PIP2 phosphatidylinositol-4,5-bisphosphate, PIP3 phosphatidylinositol-3,4,5-trisphosphate, RTK receptor tyrosine kinase, GPCR G protein-coupled receptor, MEK mitogen-activated extracellular signal-regulated kinase, ERK extracellular regulated protein kinases, TSC1/2 tuberous sclerosis complex 1/2, mTORC1/2 mechanistic target of rapamycin kinase 1/2, 4E-BP1 4E binding protein 1, EIF4e eukaryotic initiation factor 4E proteins, GSK3 glycogen synthase kinase 3, IκB inhibitor of kappa B kinase, MDM2 murine double minute 2, Casp9 caspase-9, Bcl B-cell lymphoma 2, BAD Bcl-2 antagonist of cell death

The phosphatidylinositol 3-kinase (PI3K) pathway, an evolutionarily conserved signaling axis that governs cellular survival, proliferation, and metabolic adaptation, operates through lipid-mediated signal transduction (Fig. 7).^243,244^ Specifically, membrane receptors, including receptor tyrosine kinases (RTKs), G-protein-coupled receptors (GPCRs), Toll-like receptors (TCRs), and B-cell receptors (BCRs), activate PI3K to catalyze the conversion of phosphatidylinositol-4,5-bisphosphate (PIP2) to phosphatidylinositol-3,4,5-trisphosphate (PIP3), generating a lipid second messenger that recruits AKT to the plasma membrane. AKT orchestrates oncogenic reprogramming via phosphorylation cascades: BAD inactivation suppresses apoptosis, GSK3β inhibition promotes cell cycle progression, and mTOR activation stimulates biosynthetic processes. This pathway is most frequently aberrantly activated in human malignancies and their precursor lesions,^243^ spanning breast,^245^ laryngeal,^246^ colorectal,^247^ cervical,^248^ and non-Hodgkin lymphoma.^249^ Pathogen-driven PI3K/AKT dysregulation exemplifies its carcinogenic potential: oncogenic HPV elevates p-AKT (Ser473) or p-Src (Tyr527) to initiate cervical carcinogenesis.^248^ Therapeutically, mTOR inhibition extends lifespan across preclinical models,^250^ highlighting its dual role in aging-associated precancer control and longevity regulation.

PI3K pathway dysregulation in precancers is partly attributed to oncogenic kinase activation (*PIK3CA*/*PIK3CB* mutations) coupled with inactivation of the tumor suppressor PTEN. The catalytic subunit p110α (encoded by *PIK3CA*) drives class 1 A PI3K activity, with *PIK3CA* mutations emerging as the most commonly mutated oncogene detected across tumor lineages.^251^ In precancerous contexts, these mutations manifest in endometriosis-associated ovarian lesions,^252^ the colorectal adenoma‒carcinoma transition,^253^ and high-risk laryngeal dysplasia.^246^ *PIK3CA* amplification independently initiates left-sided colorectal adenomas irrespective of *KRAS* status.^247^ *PTEN* loss, through impaired PIP3-to-PIP2 conversion,^254^ serves as an early diagnostic marker in endometrial precancers.^255–263^ Overall, mutations in the gene encoding PI3K and inactivation of PTEN converge on PIP3 accumulation, amplifying downstream oncogenic signaling. However, suitable targeting biomarkers remain challenging: while short-term alpelisib (a PI3Kα inhibitor) prophylaxis slows early lesion expansion and prevents cancer development,^245^ clinical translation is hindered by metabolic toxicity (hyperglycemia-induced insulin feedback) and compensatory resistance.^264^ Our preclinical studies demonstrated that DNMT1 inhibition outperforms PI3K/CDK inhibitors in oral cancer models by circumventing glucose dysregulation and enhancing therapeutic efficacy.^170^ These findings underscore the need for innovative strategies, such as isoform-selective inhibitors or epigenetic combination therapies, to safely disrupt PI3K signaling while mitigating resistance in precancerous contexts.

### MAPK signaling pathway

The RAS/mitogen-activated protein kinase (MAPK) signaling cascade has emerged as a pivotal oncogenic driver, with *KRAS* mutations occurring in ~25% of human malignancies.^265^ This pathway’s centrality in premalignant evolution is evidenced by *Kras*-driven transgenic models spanning pulmonary,^266^ pancreatic,^147,214^ gastric,^267^ colorectal,^268^ esophageal,^45^ and malignant cholangiocytes.^269^ For example, the *Gif-rtTA;TetO-Cre;Kras*^*G12D*^ mouse model has carcinogenic potential; doxycycline-inducible Kras activation in gastric chief cells alone is sufficient to initiate multistep carcinogenesis through premalignant metaplasia‒dysplasia transitions,^134^ underscoring the autonomous transformation capacity of proto-oncogenes within defined cellular compartments.

The MAPK signaling cascade serves as a critical transduction hub, converting extracellular stimuli into diverse cellular responses through a conserved three-tiered kinase architecture.^270^ This hierarchical system initiates with MAPKKKs responding to stimuli, which phosphorylate/activate MAPKKs, subsequently triggering MAPKs (ERK1/2, p38, JNK, and ERK5) that regulate hundreds of substrates to determine cell fate.^271^ In precancerous contexts, ERK signaling promotes stemness by overriding replication stress responses—an effect reversible through MEK/ERK inhibition that reinstates oncogene-induced senescence via MDM2/p21 axis reactivation.^272^ Pathogenetically, MAPK activation underlies metaplastic transitions preceding malignancy. Gastric intestinal metaplasia involves MAPK/oxidative phosphorylation pathway enrichment,^273^ while ERK-mediated residualization of KRT7+ transitional epithelia at squamocolumnar junctions mechanistically links MAPK signaling to Barrett’s esophagus metaplasia.^274^ Therapeutically, targeting KRAS/ERK has emerged as a strategic approach, given the prevalence of *KRAS* mutations in precancers.^269^ Although direct KRAS inhibition remains challenging due to structural undruggability,^265^ downstream ERK blockade shows promise, as exemplified by ERK1/2 inhibitors disrupting mutant *KRAS*-driven premalignant progression across multiple organ systems.

### EGFR signaling pathway

The epidermal growth factor receptor (EGFR, also known as ErbB1 or HER1), a prototypical RTK, orchestrates environmental growth signals into proliferative and survival responses through its transmembrane architecture and cytoplasmic kinase domain.^275^ The selective ligands include EGF, transforming growth factor-α, and heparin-binding EGF-like growth factor, among others. EGFR activation is initiated via ligand-induced dimerization—either homodimeric (EGFR-EGFR) or heterodimeric (e.g., EGFR-HER2) configurations—triggering autophosphorylation and downstream effector recruitment.^276^

*EGFR* is one of the most frequently mutated oncogenes in cancer and preneoplastic lesions of the oral cavity, lung, cervical tract, and gastrointestinal tract.^275,277–281^ The overexpression of EGFR and certain oncogenic mutations lead to spontaneous dimerization of the receptor, resulting in receptor activation.^282^ Inflammation-associated pEGFR-ERBB2 heterodimer upregulation in the gastric mucosa.^283^ Topically delivered molecular-specific contrast agents, which are based on anti-EGFR antibodies, have been used in vivo for the molecular imaging of precancers.^284^ Activated EGFR-mediated endosomal signaling can be sustained through the RAS/MAPK and PI3K/mTOR cascades.^285^ The heat shock factor 1 pathway has also been confirmed to be activated in parallel with EGFR in pancreatic precancerous lesions.^286^ These pathways transmit mitogenic signals to the nucleus by regulating several transcription factors, thereby controlling the expression of genes related to cellular responses, including DNA repair, proliferation, migration, differentiation, apoptosis, and inflammation.^275^ Macrophage EGFR signaling amplifies innate immune activation.^287^ Genetic attenuation of EFGR activation in mice leads to decreased levels of DNA damage in vivo during *H. pylori* infection.^283^ Myeloid-specific EGFR ablation reduces colitis-associated tumorigenesis and protects against high-grade dysplasia.^287^ Consequently, this dual role—sustaining premalignant cell survival while fueling inflammation—positions EGFR as a biomarker and therapeutic target across precancers.

### NF-κB signaling pathway

**Fig. 8:**
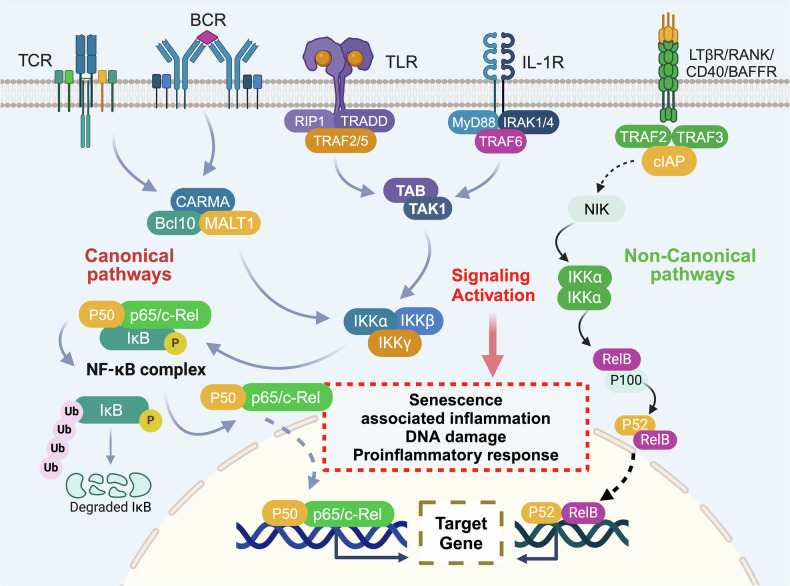
NF-κB signaling pathway in precancers. NF-κB signaling is activated in precancerous lesions and early carcinogenesis, driving inflammation (including senescence-associated inflammation). The canonical pathway is activated by pro-inflammatory stimuli and requires IKK complex activation. IKKβ phosphorylates IκB, triggering its ubiquitination and proteasomal degradation, thereby releasing nuclear-translocating NF-κB dimers (e.g., p50/c-Rel) to induce target gene transcription. The noncanonical pathway activates p52/RelB dimers via NIK stabilization and IKKα activation. IKKα phosphorylates p100, enabling its processing to p52 (not degradation). The p52/RelB dimer then translocates to the nucleus. CARMA Caspase recruitment domain (CARD)-containing membrane-associated guanylate kinase-like protein, MALT1 mucosa-associated lymphoid tissue lymphoma translocation protein 1, IκB inhibitory of NF-κB, TAB TGF-β-activated kinase 1 (TAK1)-binding protein, TAK1 TGF-β-activated kinase 1, IKKα/β/γ IκB kinase α/β/γ, NIK NF-κB-inducing kinase

The NF-κB signaling cascade operates as a molecular moderator that balances inflammation and tumorigenesis during precancerous evolution. Canonical activation involves cytoplasmic sequestration of NF-κB/inhibitor of κB (IκB) complexes, with stimulus-induced IκB kinase (IKK)-mediated phosphorylation triggering proteasomal degradation of IκB. This liberates NF-κB dimers (e.g., p50/p65) for nuclear translocation and κB enhancer binding, initiating transcriptional programs critical for premalignant progression (Fig. 8).^288^ The mechanistic drivers in precancers include Toll-like receptor (TLR)-mediated activation, the pathogen interface, and the senescence‒inflammation axis.

Oral carcinogenesis involves early NF-κB activation,^289^ with TLR2 dyslocalization (entire epithelial layers vs. basal layer-restricted) distinguishing hyperorthokeratosis or dysplasia from normal mucosa in proliferative verrucous leukoplakia.^290^ TLR4-driven COX-2 induction in Barrett’s esophagus exemplifies microbial pattern recognition receptor (PRR) synergy with inflammation-induced malignant transformation.^291^ The NF-κB pathway is also closely associated with the pathogen response. *H. pylori* susceptibility in GPLs involves genetic mechanisms (*NFKB1*/rs1598861 and rs7674640, *TLR1*/rs4833095, *TLR10*/rs10004195, and *TLR9*/rs5743836 and rs187084).^156,292,293^ *H. pylori* coopts NF-κB through virulence factor (CagA)-mediated mechanisms, which induce IL-6/IL-17A crosstalk between immune cells and the gastric epithelium.^294^ In addition, as previously mentioned, the SASP and inflammaging are closely linked to precancers. The NF-κB signaling pathway integrates the intracellular regulation of immune responses, aging, and age-related diseases, making it the main culprit of inflammaging.^295^ NF-κB integrates SASP regulation and inflammaging via p65-mediated senescence enhancement and CXCR2-driven epithelial aging in *H. pylori* infection,^84,296^ whereas PAR-2/NF-κB and KLF7/NF-κB coupling elicits inflammation in precancers.^297,298^

Although NF-κB inhibition attenuates precancerous phenotypes (e.g., bortezomib reduces prostatic intraepithelial neoplasia formation in *Foxp3*-mutant mice),^299^ complete pathway ablation via the deletion of NF-κB essential modulator paradoxically accelerates pancreatic cancer progression and reduces the median lifespan despite reducing the early PanIN burden in young mice.^300^ This duality underscores the need for context-stratified targeting strategies that preserve tumor-suppressive senescence while inhibiting procarcinogenic inflammation.

### JAK/STAT signaling pathway

The Janus kinase-signal transducer and activator of transcription (JAK-STAT) pathway is a pivotal molecular conduit linking chronic inflammation to premalignant progression.^301^ Originally characterized in interferon responses,^302^ this pathway enables rapid signal transduction from membrane receptors to nuclear transcription, positioning it as a master regulator of inflammatory carcinogenesis.^301^ The JAK-STAT pathway regulates proinflammatory cytokines, such as IL-6, IL-10, and TNF-α.^303^

*H. pylori* rapidly triggers STAT3/COX-2; gastric STAT3 signaling induces COX-2, which amplifies IL-6/STAT3 activity during infection.^304^ Deoxycholic acid, the main bile acid component of duodenogastric reflux, sustains STAT3-KLF5 activation during the gastritis-to-intestinal metaplasia transition.^305^ JNK/p38-dependent STAT3 activation in colon epithelia upregulates CD80 to induce ROS-mediated immune evasion.^306^ During acinar-to-ductal metaplasia and early PanIN lesions, the IL17-REG3β-JAK2/STAT3 axis promotes cell growth and decreases sensitivity to cell death.^307^ Moreover, high pSTAT3 (Tyr705) levels are correlated with intestinal metaplasia and dysplasia.^304^ pSTAT3-mediated VEGF overexpression accelerates the gastritis-to-cancer transition,^308^ whereas a concomitant gain of pSTAT3 (Tyr705) and loss of a protein inhibitor of activated STAT3 enable high-grade serous carcinogenesis.^309^ These studies offer ample examples of the overexpression and hyperactivation of STAT3 signaling associated with the malignant transformation of precancers. Given the therapeutic effect of relevant ongoing clinical trials for STAT3 inhibitors in cancer treatment,^310^ the potential for STAT3-targeted interventions in precancerous diseases warrants further exploration.

### Hippo signaling pathway

The Hippo pathway functions as a central regulator of tissue homeostasis, coordinating a conserved kinase cascade from the tumor suppressors MST1/2 to the oncoproteins YAP/TAZ to balance cell proliferation and apoptosis.^311^ While Hippo pathway dysregulation induces YAP/TAZ-driven overexpression of prosurvival genes (e.g., c-MYC and survivin) and tumorigenesis in model organisms,^312,313^ human cancers rarely exhibit Hippo gene mutations, suggesting that the oncogenic role of the Hippo pathway is predominantly driven by microenvironmental dysregulation.

In host‒pathogen interactions, Hippo activation exerts protective effects by controlling epithelial homeostasis: during *H. pylori* infection, LATS2-mediated YAP1 phosphorylation suppresses infection-induced metaplasia and preserves gastric epithelial identity,^314^ whereas obesity-associated JCAD impairs phospho-YAP/YAP ratios in nonalcoholic steatohepatitis-precancerous lesions, disrupting hepatic homeostasis.^315^ Paradoxically, aberrant YAP/TAZ activation synergizes with intrinsic signals (e.g., NRG1) to disrupt airway epithelial polarity^316^ and is correlated with precancerous lesion severity in breast cancer and lung squamous carcinoma.^316,317^ Survivin upregulation across the gastritis-intestinal metaplasia-dysplasia-adenocarcinoma continuum underscores YAP-dependent oncogenicity.^318^ This functional duality arises from the dependency of YAP/TAZ on secondary signals (e.g., additional mutational events and inflammation) to transition from homeostatic repair to progrowth states. Mutational cooperativity further drives transformation, exemplified by GNAS^R201C^ collaborating with oncogenic KRAS^G12V/D^ to sequester phosphorylated YAP1 cytoplasmically.^319^ In bronchial premalignancy, the YAP/TAZ-TP63 complexes repress interferon responses to enable basal cell hyperproliferation.^320^ Chronic inflammatory milieus coopt Hippo effectors to promote precancerous development.^321^ The extracellular matrix protein periostin from fibroblasts promotes YAP/TAZ nuclear localization and IL-6 expression to ultimately facilitate colorectal precancerous development.^322^ The murine liver-specific deletion of MST1/2 led to massive YAP-mediated macrophage infiltration, which sustained a protumor microenvironment, which was reversed by YAP ablation.^323^ The contradictory role of the Hippo pathway may be related to the fact that YAP activation alone is insufficient to license its oncogenic potential in precancerous lesions without this additional signal.^324^ Therapeutic strategies must therefore balance Hippo’s tumor-suppressive functions against its coopted oncogenic potential in inflammatory contexts.

### Other signaling pathways

In addition to major signaling cascades, several additional pathways contribute to precancerous progression through their roles in epithelial homeostasis, angiogenesis, and microenvironmental adaptation. The cyclic nucleotides, such as cyclic guanosine monophosphate (cGMP) and cyclic adenosine monophosphate (cAMP) are considered ubiquitous second messengers.^325^ Targeting cGMP with either phosphodiesterase-5 inhibitors or receptor guanylyl-cyclase C agonists alters epithelial homeostasis in a manner that reduces neoplasia.^326^ The Hedgehog pathway drives angiogenic and tissue remodeling programs in oral precancers, as evidenced by upregulated Hedgehog protein expression in clinical biopsies.^327,328^ Hypoxia-related signaling further links microenvironmental stress to malignant transformation: carbonic anhydrase IX overexpression in gastroesophageal reflux disease creates an acidic environment conducive to esophageal tumorigenesis,^329^ whereas hepatocyte-derived HIF-1α activation during nonalcoholic fatty liver disease progression adapts cells to hypoxic stress, promoting metabolic reprogramming and angiogenesis.^330^

These pathways collectively shape the “degenerated soil” of precancerous microenvironments through epithelial dysregulation, vascular remodeling, and hypoxia-driven adaptation. Targeting these signaling hubs offers a strategic approach to intercepting the crosstalk between precancerous “seeds” and their permissive microenvironments, presenting a promising frontier for therapeutic innovation.

## Signaling pathway- and biomarker-based intervention strategies

Advances in biological understanding and the accelerating pace of technological progress are driving rapid evolution in precancer intervention strategies. Despite significant progress in anticancer therapies, most advanced solid tumors are still incurable. Active precancer intervention can eliminate the potential for tumorigenesis, sparing patients from complex open surgeries and long-term recovery processes. Current clinical management of high-risk precancerous states remains anchored in empiric pharmacotherapy, highlighting a critical gap in molecularly targeted prevention. This section synthesizes emerging therapeutic strategies informed by the preceding pathway analyses, proposing a framework for translating mechanistic insights into precision interception of premalignant progression.

### Targeting macroenvironmental drivers

#### Senolytics and senomorphics

Despite robust evidence linking aging to precancerous progression, senotherapies, including senolytics (selective elimination of senescent cells) and senomorphics (inhibition or modulation of SASP),^331^ remain underexplored in clinical precancer management. Emerging candidates for senolytic strategies for precancer interception include the BCL-2 family inhibitor Navitoclax to inhibit autophagy,^332^ dasatinib combined with quercetin for targeting RTKs and the PI3K/mTOR pathways,^333,334^ and CAR-T cells which target urokinase plasminogen activator receptor (uPAR) on the surface of senescent cells,^335^ have shown promise in cancer and other disease models. However, the dual role of senescence (tumor-suppressive vs. proinflammatory) necessitates cell type-specific targeting to avoid eliminating protective senescent populations. In contrast, SASP modulation offers safer therapeutic windows by neutralizing oncogenic secretions.^336^ The mTOR inhibitor rapamycin is an example of drug repurposing. As a selective SASP inhibitor, it has been shown to extend both lifespan and health span^337^ and reduce the protumorigenic potential of senescent cells in vivo.^338,339^ Metformin, one of the most prominent repurposed drugs, has demonstrated multifaceted antitumor activity and clinical risk control.^340,341^ Metformin can effectively inhibit precancerous progression to invasive cancer by blocking STAT3 activation,^342^ mutated PIK3CA and HPV oncogene-induced mTOR signaling,^343^ and the nuclear translocation of the NF-κB pathway.^344^ Metformin also reduces the expression of iNOS, COX-2, and IL-6.^345^ Metformin’s ability to attenuate SASP expression and impede premalignant progression in bladder cancer and HNSCC models underscores its potential as a precision senomorphic agent.^344,346^ Metformin efficacy is correlated with STK11/LKB1 mutations and HPV/PIK3CA alterations,^340^ suggesting biomarker-driven application. These developments underscore the potential of senotherapy to disrupt the senescence‒inflammation‒cancer axis, although precision targeting remains critical to harness senescence biology safely for precancer interception.

#### Microbiome-based interventions

The human microbiome represents a promising therapeutic target for precancerous lesions with established microbial etiologies, such as CINs and GPLs. The HPV vaccine exemplifies successful microbiome-based prevention, significantly reducing HPV-related infections and precancers (Table 2).^347^ Similarly, *H. pylori* eradication has demonstrated clinical efficacy in GPL management^103,348,349^ and even reverses the progression of GPLs with epigenetic alterations and milder lesions.^350^ However, persistent gastric mucosal methylation after eradication may increase the risk of metachronous cancer.^351^ These findings underscore the need for combinatorial approaches that integrate microbiome modulation with other therapies to optimize precancer interventions. Panprecancerous strategies focus on augmenting host immunity through exogenous microbiota manipulation, including probiotics and microbiota transplantation. Probiotic use has demonstrated significant antimutagenic effects on the development of colon preneoplastic lesions in rats.^352^ Microbiota transplantation is an approach that involves the administration of microbiota from healthy donors into patients, including fecal microbiota transplantation (FMT).^353^ FMT restores microbial diversity and enhances immune responses. FMT from anti-PD-1 responders improves checkpoint inhibitor efficacy in refractory solid tumors.^354^ While microbiota-based therapies remain underexplored in precancer management, their potential to reshape immune‒microbe interactions offers a novel paradigm for early intervention. Future research should consider additional microbiome profiling methods to identify predictive biomarkers and optimize therapeutic microbial consortia for precision prevention.

**Table 2:** Clinical trial for precancer treatment

Treatment	Disease	Target	Outcome	Phase; Status	Registration number	Refs
Vaccine						
HPV-16/18 AS04-adjuvanted vaccine	CIN2+	-	High efficacy against HPV16/18-associated precancer	Phase 3; Completed	NCT00128661	^483^
	CIN2 + , CIN3+	-	Long-term absolute reductions in high-grade lesions	Observational; Active	NCT00867464	^484^
VGX-3100	CIN2/3, CIN3	Therapeutic vaccine targeting E6/E7	Efficacy against CIN2/3 associated with HPV16/18	Phase 2b; Completed	NCT01304524	^50,485^
GX-188E	CIN2, CIN2/3, CIN3	Therapeutic vaccine targeting E6/E7	No results posted	Phase 2; Unknown status	NCT02596243	Unpublished
KMTLL16E6/7	CIN2/3	Therapeutic vaccine targeting E6/E7	No results posted	Phase 2; Recruiting	IRCT20190504043464N2	Unpublished
Monoclonal antibody						
Nivolumab	OPVL	PD-1	Potential clinical activity for nivolumab in high-risk OPVL	Phase 2; Active	NCT03692325	^51^
Pembrolizumab	OLK	PD-1	No results posted	Phase 2; Recruiting	NCT03603223	Unpublished
Pembrolizumab	High-risk IPNs	PD-1	No results posted	Phase 2; Recruiting	NCT03634241	Unpublished
Atezolizumab+ Bevacizumab	Liver cirrhosis	PD-L1 and VEGF	No results posted	Phase 2; Recruiting	NCT06096779	Unpublished
Nivolumab+JNJ-73763989+ Nucleos(t)ide Analogs	Chronic Hepatitis B	PD-1	No results posted	Phase 2; Recruiting	EUCTR2021-005132-33-ES	Unpublished
Small-molecule inhibitor						
PRI-724 (OP-724)	HCV-induced liver cirrhosis	CBP/β-catenin inhibitor	Improvement of liver histology and Child Pugh class	Phase 1; Completed	NCT02195440	^376^
	HCV/HBV-derived liver cirrhosis	CBP/β-catenin inhibitor	Improvements of liver stiffness	Phase 1, 2; Completed	NCT03620474	^377^
	Primary biliary cholangitis	CBP/β-catenin inhibitor	Well-tolerated	Phase 1; Completed	NCT04047160	^378^
	Liver cirrhosis	CBP/β-catenin inhibitor	Well-tolerated	Phase 1; Completed	NCT04688034	^486^
	Liver cirrhosis	CBP/β-catenin inhibitor	No results posted	Phase 2; Recruiting	NCT06144086	Unpublished
Baricitinib	OLP	JAK-1/2 inhibitor	No results posted	Phase 2; Recruiting	NCT06158113	Unpublished
	Hepatocellular adenomas	JAK-1/2 inhibitor	No results posted	Phase 2;Not yet recruiting	NCT06490757	Unpublished
AVX001	AK	cPLA2α inhibitor	Potential treatment option	Phase 1, phase 2; Unknown status	NCT05164393	^487,488^
Tirbanibulin	AK	Tubulin polymerization and Src kinase inhibitor	Superior to placebo but related to transient reactions and lesion recurrence at 1 year	Phase 3; Completed	NCT03285477; NCT03285490	^489^
Gefitinib	DCIS	EGFR inhibitor	No results posted	Phase 2; Terminated	NCT00082667	Unpublished
Erlotinib	Liver cirrhosis	EGFR inhibitor	No results posted	Phase 2; Not yet recruiting	NCT04172779	Unpublished
Imiquimod	HSIL	TLR7 agonist	Reducing progression to cancer	Phase 3; Completed	NCT02135419	^490^
	Residual/recurrent CIN		Therapeutic resistance	Phase 3; Unknown status	NCT02669459	^491^
	CIN 2/3		Non-invasive yet less effective than LLETZ	Phase 3; Unknown status	NCT02917746	^492^
Repurposed drugs						
Metformin	OLK; Erythroplakia	PI3K/mTOR	Partial or complete histological response	Phase 2; Active	NCT02581137	^493^
	OLK; Erythroplakia	pS6, nuclear YAP	No results posted	Phase 2; Active	NCT05237960	Unpublished
Celecoxib	OLK; Dysplasia	COX-2 inhibitor	No results posted	Phase 2; Completed	NCT00052611	Unpublished
HCQ	Severe erosive OLP	HSP90β agonist	Efficacy for treatment	Phase 4; Completed	ChiCTR2100042577	^400,494^
Thalidomide	Erosive OLP	TNF-α inhibitor	Efficacy for treatment	Phase 4; Completed	-	^495^
Tacrolimus	DLE	Calcineurin inhibitor	Efficacy for treatment	Phase 4; Completed	ChiCTR-TRC-11001195	^496^
Tamoxifen	ADH, DCIS, LCIS	ERs modulator	Preventing recurrence of breast intraepithelial neoplasia	Phase 3; Active	NCT01357772	^497–499^
	MCN	ERs modulator	No results posted	Phase 1; Recruiting	NCT06320990	Unpublished
Artesunate	CIN2/3	Inhibitor of STAT3 and exported protein 1	No results posted	Phase 1; Recruiting Phase 2; Recruiting	NCT06165614; NCT06519994	Unpublished
Ingenol Mebutate	AK	PKCα, PKCδ agonist	Efficacy for field treatment	Phase 3; Completed	NCT00742391;NCT00916006;NCT00915551; NCT00942604	^500^
Fluorouracil	AK	TYMS inhibitor	Efficacy for treatment	Phase 4; Unknown status	NCT02281682	^501–504^
Tuvatexib	AK	HK2/VDAC1 regulator	No significant difference compared to placebo	Phase 2; Completed	NCT02844777	Unpublished

*ADH* atypical ductal hyperplasia, *AK* actinic keratoses, *CBP* CREB-binding protein, *CIN* cervical intraepithelial neoplasia, *COX-2* cyclooxygenase-2, *cPLA2α* cytosolic phospholipase A2α, *DCIS* ductal carcinoma in situ, *DLE* discoid lupus erythematosus, *EGFR* epidermal growth factor receptor, *ER* estrogen receptor, *HBV* hepatitis B virus, *HCV* hepatitis C virus, *HCQ* hydroxychloroquine, *HK2* hexokinase 2, *HPV* human papillomavirus, *HSP90α/β* heat shock protein 90 alpha or beta, *HSIL* high-grade squamous intraepithelial lesion, *LLETZ* large loop excision of the transformation zone, *IPMN* intraductal papillary mucinous neoplasms, *IPN* indeterminate pulmonary nodules, *JAK* Janus kinase, *LCIS* lobular carcinoma in situ; *MCN* pancreatic mucinous cystic neoplasms, *OLK* oral leukoplakia, *OLP* oral lichen planus, *OPMD* oral potentially malignant disorders, *OPVL* oral Proliferative verrucous leukoplakia, *PD-1* programmed cell death protein 1, *PD-L1* programmed cell death protein 1 ligand, *PDE4* phosphodiesterase 4, *PI3K/mTOR* phosphatidylinositol 3-kinase/mammalian target of rapamycin, *PKC* protein kinase C, *pS6* phospho-S6 ribosomal protein, *RNR* ribonucleotide reductase, *TKI* tyrosine kinase inhibitors, *TLR7* toll like receptor 7, *TNF-α* tumor necrosis factor alpha, *TYMS* thymidylate synthase, *VDAC1* voltage dependent anion channel 1, *VEGF* vascular endothelial growth factor, *YAP* Yes-associated protein, *NA* not applicable

#### Metabolic and dietary interventions

The pivotal role of metabolic reprogramming in shaping oncogenic phenotypes during precancerous progression offers novel opportunities for precision screening and therapeutic targeting.^355,356^ While conventional antifolates (e.g., methotrexate and pemetrexed) remain cornerstone agents in chemotherapy because they suppress tumor-associated nucleotide biosynthesis,^357^ their clinical utility is limited by off-target toxicity in proliferative tissues. In contrast, emerging metabolic modulators, including nutritional intervention and metabolic pathway recalibration, demonstrate enhanced safety profiles for precancerous intervention.

Dietary strategies show promise in early interventions. Combinatorial administration of fish oil with tamoxifen achieves prevention and regression of mammary precancers.^358,359^ Preclinical studies have revealed that fidarestat, an aldose reductase inhibitor, mitigates obesity-driven colonic premalignancy through polyol pathway blockade.^360^ Olive oil supplementation reduces colorectal carcinogenesis in rats via arachidonic acid (AA) metabolic rewiring,^361^ which is particularly relevant given early AA pathway activation in familial adenomatous polyposis hyperplasia.^362^ Pharmacological targeting of AA derivatives has translational potential: Montelukast, a cysteinyl leukotriene receptor antagonist, suppresses colonic epithelial hyperproliferation through leukotriene B4 receptor antagonism.^363,364^ All-trans retinoic acid (ATRA) supplementation exerts anti-neoplastic effects in gastric precancerous lesions (GPLs)^365^ through suppression of malignant transformation via exosomal LncHOXA10/pyruvate carboxylase axis modulation.^366,367^ Moreover, phytomedicines exhibit multitarget regulatory capacities: asarinin induces mitochondrial ROS-mediated STAT3 inactivation,^368^ whereas Sijunzi decoction ameliorates gastric precancerous lesions by regulating oxidative phosphorylation and inhibiting the HIF-1α signaling pathway.^369^ In addition, the 2017 FDA approval of enasidenib (IDH2 inhibitor) for AML treatment marked a paradigm shift in cancer metabolism therapeutics.^370^ Notably, the IDH1/2 mutational landscape in precancers remains unexplored—a knowledge gap that may unveil a novel metabolism-specific prevention strategy.

### Modulating epithelial cell fate and survival

As previously mentioned, the plasticity of epithelial cell fate in precancerous evolution is governed by dynamic crosstalk between mutational drivers (e.g., TP53, EGFR, and Wnt) and their signaling networks. Novel hydantoin-based dispiroindolinones demonstrate allosteric inhibition of p53-MDM2 binding, whereas optimized Passerini caspase activators induce p53-dependent apoptotic priming through conformational stabilization.^371^ EGFR tyrosine kinase inhibitors (TKIs) exert dual cytostatic effects on premalignant breast epithelium by suppressing proliferation and enhancing apoptosis.^372^ Clinical studies have revealed that cetuximab-mediated histopathological regression occurs in aerodigestive tract dysplasia,^373^ whereas the chemopreventive efficacy of aspirin is correlated with EGFR expression homeostasis in patients with colorectal premalignancy.^374^ Among the Wnt pathway components, nuclear β-catenin/CBP transcriptional complexes represent druggable nodes for epigenetic reprogramming. PRI-724 achieves CBP-selective β-catenin dissociation, preserving P300-mediated homeostatic signaling while attenuating fibrotic progression in cirrhotic models.^375–378^ Pyrvinium plays a role in β-catenin suppression reprogramming the PME^379^ and enhancing the proapoptotic Bcl-2 family inhibitor ABT263-induced apoptosis in colon carcinogenesis.^380^

Additionally, botanical agents have pleiotropic effects on epithelial homeostasis, including cell cycle modulation, apoptotic reprogramming, and EMT inhibition. Astragaloside IV prevents gastric metaplasia by cyanidin-3-O-glucoside induces G1/S phase arrest in hepatocarcinogenesis models.^381^ The Zuojin capsule coordinates PI3K-AKT-Bcl2 axis suppression with cell cycle kinase inactivation.^382^ Gallic acid, Moluodan, and ginsenoside Rb1 antagonize Wnt/β-catenin-driven EMT through β-catenin/E-cadherin axis stabilization.^239,383,384^ With the rapid advancements in chemistry and medical technology, the integration of phytochemical libraries with CRISPR-Cas9 high-throughput screening platforms may accelerate the discovery of stage-specific epithelial modulators, particularly for lesions exhibiting concurrent pathway coactivation.

### Disruption of microenvironmental remodeling

#### Immunotherapies

While checkpoint inhibitors (ICIs) demonstrate established efficacy in advanced malignancies, their spatiotemporal application in premalignancy requires precision modulation. Among these, anti-PD-1 antibodies have revolutionized the management of many precancers. Prophylactic administration of polyinosinic-polycytidylic acid (polyIC) during premalignant stages sensitizes the liver to PD-L1 blockade by reprogramming hepatic immunosurveillance—activating natural killer cells and polarizing macrophages—thereby suppressing tumorigenesis in murine hepatocarcinogenesis models.^385^ In cirrhotic liver models, anti-PD-1 mAbs prevent HCC progression by enhancing CD8 + T-cell parenchymal infiltration without compromising hepatic synthetic function.^386^ Murine oral carcinogenesis studies have demonstrated dual-phase cytokine modulation—early IL-6/IL-17/TNF-α surge (week 1) followed by sustained IFN-γ elevation (week 3)—correlated with the inhibition of precancer to oral cancer.^387,388^ PD-1 inhibition is also accompanied by STAT1 activation and the production of the T-cell effector granzyme B in infiltrating cells and by the induction of apoptosis in the epithelial cells of oral lesions,^389^ suggesting T-cell–mediated immunoprevention. Importantly, treated mice exhibit no overt immune-related toxicities,^386^ supporting the feasibility of safe dosing regimens.

#### Anti-inflammatory therapies

Chronic inflammation and the SASP form a self-reinforcing loop in age-related precancerous evolution, with shared molecular nodes (e.g., NF-κB) offering dual therapeutic targeting opportunities. Additionally, some inhibitors of the TGF-β, STAT, and other proinflammatory pathways have biological activities in precancer interventions. TGF-β/IL-23 axis inhibition in murine oral premalignancy models induces the upregulation of splenic immunoregulatory mediators, coupled with a reduction in lesion progression rates.^390^ Dual p38/STAT3 pathway blockade by tanshinone I reverses EMT and resolves inflammation in GPLs.^391^ Phytomedicine manpixiao decoction orchestrates multicytokine normalization in GPL rats, which is correlated with a decrease in the risk of gastric cancer.^392^ NF-κB signaling pathway-related molecules can also be regulated by Weifuchun tablets (WFCs) in gastric intestinal metaplasia and dysplasia.^393^ However, the spectrum of activity and potential effects for these natural products should be expanded because many of them have been tested on a limited number of precancerous and malignant types. Future directions should integrate multiomics platforms to map inflammation‒senescence interactomes across precancer stages and repurpose present agents via pharmacodynamic biomarkers.

#### Antiangiogenic therapies

The most widely used antiangiogenic agents include tyrosine kinase inhibitors (TKIs) that target the vascular endothelial growth factor (VEGF) pathway. Sunitinib demonstrates selective vulnerability in neoplastic Barrett’s epithelium through VEGF/VEGFR2 inhibition.^394^ Atractylenolide III suppresses the HIF-1α/VEGF-A/DLL4 axis, reducing gastric premalignant lesions in rat models via pericyte detachment and vascular normalization.^395^ Additionally, precise information on localized variations in blood circulation can be detected by narrow band imaging^396,397^ and a blood perfusion imager.^398^ Quantitative estimates of blood perfusion intrinsically reflect changes in tissue and local lesion metabolism.^398^ The feeding artery feature in contrast-enhanced ultrasound is a valuable feature for distinguishing small hepatocellular carcinoma lesions from precancerous lesions,^399^ implying that localized variations in blood circulation could provide a novel noninvasive in situ screening approach for distinguishing precancer, cancer, and normal scenarios.

#### Therapeutic resistance

One of the underlying assumptions for the advantages of early interventions in precancer patients is that premalignant cells are less mutated and heterogeneous than in advanced cancer patients are, making precancerous lesions more responsive to targeted therapies. However, therapeutic resistance remains a significant challenge, manifesting in both nontargeted and targeted treatment modalities. In the context of nontargeted therapies, well-documented cases include glucocorticoid resistance.^400^ With respect to targeted therapies, emerging evidence from an oral precancerous disease trial revealed that patients exhibiting somatic copy number loss at 9p21.3 in pretreatment biopsies demonstrated resistance to ICIs.^51^ These patients subsequently progress to oral cancer following anti-PD-1 therapy. The mechanistic basis for this resistance lies in the genetic alterations at chromosome 9p. The 9p21 locus is particularly significant, as focal loss of heterozygosity events in this region are characteristic of oral precancers^188^ and chromosome 9p loss have been established as predictive biomarkers for ICI resistance.^401–403^ This genomic region represents a critical immune regulatory hub, with larger deletions frequently encompassing key immune-modulatory genes, including *CD274* (encoding PD-L1) at 9p24.1, *JAK2* at 9p24.1, and the complete interferon gene cluster at 9p21.3. These genomic alterations can drive the evolution of immune-cold oral squamous carcinoma phenotypes, thereby facilitating therapeutic resistance. Furthermore, the phenomenon of PME degeneration significantly contributes to treatment resistance. Experimental evidence from chemical carcinogenesis models in mice has demonstrated that activated fibroblasts can compromise the efficacy of PD-1 blockade through extracellular matrix remodeling.^404^ Therefore, resistance to targeted therapies for precancers is attributed to the sporadic acquisition of resistance mutations during their multiclonal formation, as well as the degeneration of the microenvironment. This comprehensive understanding of resistance mechanisms underscores the complexity of therapeutic interventions in precancerous conditions and highlights the need for multifaceted treatment strategies that address both genetic and microenvironmental factors.

**Fig. 9:**
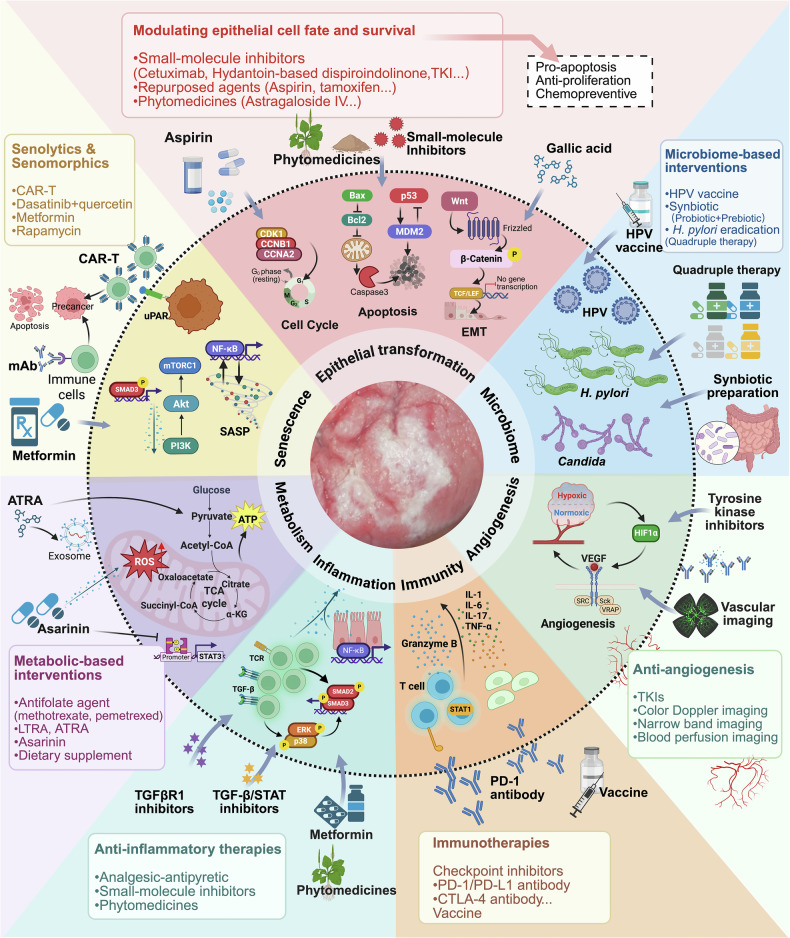
Overview of signaling pathway- and biomarker-based therapies for precancers. Targeted therapeutic strategies for precancers are primarily categorized into three mechanistic domains: (1) epithelial cell regulation through fate determination and survival modulation; (2) macroenvironmental modulation via senolytic agents, senomorphic compounds, microbiome-based interventions, and metabolic-based interventions; and (3) microenvironmental modification employing immunotherapies or anti-inflammatory and antiangiogenic agents. The key therapeutic modalities under investigation include small-molecule inhibitors, monoclonal antibodies, and vaccine-based approaches, alongside the repurposing of pharmacological agents (e.g., aspirin, metformin, and rapamycin). Additionally, emerging research has explored the potential of phytomedicines and nutraceutical supplements as adjunctive therapies in precancer management

Overall, early intervention before the onset of cancer remains a persistent challenge. Current treatment options for precancers offer a wide range of possibilities, including various drugs targeting both the epithelium and the PME, ranging from phytomedicine to small-molecule targeted therapies, and from vaccine injections to cell transplants (Fig. 9). Agents that have achieved significant success in tumor treatment, such as immune-based ICIs, CAR-T-cell therapies, TKIs targeting EGFR, small-molecule inhibitors targeting the Wnt or JAK/STAT pathways, and various drugs targeting metabolism and angiogenesis, are increasingly being applied to treat precancers (Table 2). As our understanding of these diseases deepens, the timeline for expanding targeted therapies from malignant cancers to precancerous states may shorten. However, without personalized screening, empirically using targeted inhibitors to treat precancers will inevitably result in treatment resistance in some patients. Precancerous diseases provide a critical window of opportunity to explore the most effective therapeutic strategies for optimal outcomes. To overcome challenges such as off-target effects and drug resistance, the epithelial‒mesenchymal common target (EMCT) we previously proposed could serve as a promising alternative,^213^ as stromal and immune cells within the microenvironment exhibit genetic stability.^405^ Targeting dysregulated TβRIII, which occurs simultaneously in both oral cancer cells and adjacent fibroblasts, has proven more effective than targeting cancer cells alone.^213^ Other studies have reported that the SRC family kinase LYN can serve as a common target for both the epithelial and immune microenvironments.^406^ Furthermore, the combination of anti-PD-L1 and anti-VEGF monoclonal antibodies, recently applied in viral hepatitis treatment, illustrates the potential of cotargeting immune and vascular regulatory mechanisms within the microenvironment.^407^ These findings underscore the importance of systematically addressing microenvironmental degeneration in precancerous interventions, potentially revolutionizing our approach to early cancer prevention and treatment.

## Conclusion and perspective

Over a century of scientific inquiry into precancerous states has yielded three transformative milestones: the evolution of precise terminology, breakthroughs in mechanistic understanding, and the advent of targeted therapeutic strategies. While this field holds immense potential, a unified conceptual framework for defining precancers—spanning both solid and hematologic malignancies—has only recently achieved international consensus.^2^ This framework not only establishes a critical foundation for future research but also accelerates efforts to decipher the biological continuum from the precancerous state to invasive malignancy.

Synthesizing two decades of literature, this review systematically maps the multidimensional landscape of precancer biology. We delineate the complex phenotypic spectrum of precancers, emphasizing pivotal drivers such as epithelial plasticity and dynamic macro/microenvironmental interactions. Central to this discussion is over ten dysregulated signaling pathways that orchestrate disease progression, including aging-related alterations in the p53, PI3K/mTOR, and NF-κB pathways; mutation-driven cascades within the p53, Wnt, PI3K, MAPK, and EGFR pathways; the TGF-β pathway-mediated interactions between the epithelium and the stromal microenvironment; alterations in the JAK/STAT pathway regulating inflammation; senescence-associated inflammation mediated by the NF-κB pathway; Hippo signaling pathway-mediated inflammation during host‒pathogen interactions; the role of the JAK/STAT and HIF-1 pathways in metabolic reprogramming; and angiogenesis shifting via the Hedgehog pathway. In addition to cataloging existing biomarkers and targeted therapies, this work pioneers a paradigm-shifting perspective: the microenvironment’s inductive, dominant, and even guiding roles in steering precancer evolution. We introduce the novel concept of “soil degeneration” in synergistic oncogenesis, positing that microenvironmental degradation—not merely epithelial mutations—serves as a critical catalyst for malignant transformation.

A cardinal challenge lies in defining optimal intervention windows, given the protracted timeline (years to decades) of precancer-to-cancer progression.^2^ We propose that there are two key time windows for effective intervention: before irreversible precancerous lesions develop (when interventions can still reverse the lesions) and before high-risk malignancies emerge (when the lesions remain controllable). Overemphasizing the cancer risk in precancers may lead to unnecessary interventions; therefore, stratified strategies should be applied at different stages. A systematic inhibitory strategy targeting epithelial-degenerated soil could be a promising breakthrough. For example, before irreversible lesions develop, preventive measures such as the HPV vaccine, aspirin, and dietary supplements can be employed, primarily to target degenerated soil rather than premalignant cells. Once identifiable lesions are present, advanced precancers could benefit from combination therapies addressing both epithelial mutations (e.g., EGFR inhibitors) and microenvironmental drivers (e.g., TGF-β blockers and ICIs). Targeted therapies can offer precise interventions, including organoid studies to identify personalized preventive agents and mechanisms of drug resistance, as demonstrated in Barrett’s esophagus and gastric precancers.^408–410^ However, this paradigm shift demands rigorous investigation of microenvironmental biomarkers,^411^ as changes in the PME can even induce spontaneous epithelial carcinogenesis.^211^ By integrating multiomics profiling of stromal components with epithelial genomics, we anticipate transformative advances in risk stratification and personalized prevention. Ultimately, decoding the “degenerated soil” of precancers promises to redefine oncology’s preventive frontier—shifting from reactive cancer treatment to proactive microenvironmental reprogramming for durable patient benefit.
